# Evolution of ectomycorrhizas as a driver of diversification and biogeographic patterns in the model mycorrhizal mushroom genus *Laccaria*

**DOI:** 10.1111/nph.14270

**Published:** 2016-11-07

**Authors:** Andrew W. Wilson, Kentaro Hosaka, Gregory M. Mueller

**Affiliations:** ^1^ Chicago Botanic GardenPlant Science and Conservation1000 Lake Cook RoadGlencoeIL60022USA; ^2^ Sam Mitchel Herbarium of FungiDenver Botanic Gardens909 York StreetDenverCO80206USA; ^3^ Department of BotanyNational Museum of Nature and ScienceTsukubaIbaraki305‐0005Japan

**Keywords:** biogeography, diversification, ectomycorrhiza, evolutionary ecology, molecular dating, new species, stable isotopes, systematic evolution

## Abstract

- A systematic and evolutionary ecology study of the model ectomycorrhizal (ECM) genus *Laccaria* was performed using herbarium material and field collections from over 30 countries covering its known geographic range.
- A four‐gene (nrITS, 28S, RPB2, EF1α) nucleotide sequence dataset consisting of 232 *Laccaria* specimens was analyzed phylogenetically. The resulting Global *Laccaria* dataset was used for molecular dating and estimating diversification rates in the genus. Stable isotope analysis of carbon and nitrogen was used to evaluate the origin of *Laccaria's* ECM ecology.
- In all, 116 *Laccaria* molecular species were identified, resulting in a near 50% increase in its known diversity, including the new species described herein: *Laccaria ambigua*. Molecular dating indicates that the most recent common ancestor to *Laccaria* existed in the early Paleocene (56–66 million yr ago), probably in Australasia. At this time, *Laccaria* split into two lineages: one represented by the new species *L. ambigua*, and the other reflecting a large shift in diversification that resulted in the remainder of *Laccaria*. *L. ambigua* shows a different isotopic profile than all other *Laccaria* species.
- Isotopes and diversification results suggest that the evolution of the ECM ecology was a key innovation in the evolution of *Laccaria*. Diversification shifts associated with *Laccaria's* dispersal to the northern hemisphere are attributed to adaptations to new ecological niches.

## Introduction

Ectomycorrhizal (ECM) fungi are symbiotic associates of dominant plant species found in most forest ecosystems (Smith & Read, 2008). Their ability to provide nutrients to their plant partner and engage in nutrient cycling is crucial for forest function and stability (Simard, 2009; van der Heijden *et al*., 2015). The importance of mycorrhizal fungi extends to promoting climate stability given their importance in sequestering carbon (Clemmensen *et al*., 2013).

It is believed that there is an important correlation between the ECM ecology and fungal diversity. While arbuscular mycorrhizal fungi associate with the greater proportion of terrestrial plant species (> 70% vs 2%)(Brundrett, 2009), the diversity of ECM fungal species is much greater (> 7000 vs 230) (Rinaldi *et al*., 2008). This diversity is partly the result of as many as 80 independent evolutions of the ECM ecology among Agaricomycetes (mushrooms and allies) (Tedersoo & Smith, 2013), but understanding how this ecology functions as a driver of diversification in fungal lineages continues as a subject of interest in the study of ECM fungi (Bonito *et al*., 2013; Sánchez‐Ramírez *et al*., 2015). A recent study observed that even though there is little pattern in the origin of ECM lineages, the successful ones tend to have a net positive diversification rate (Ryberg & Matheny, 2012). Despite this, there is currently no evidence that directly correlates a shift in fungal diversification rates with a switch to the ECM ecology.

The mushroom‐forming genus *Laccaria* (Basidiomycetes, Agaricomycetes, Hydnangiaceae) is a model for the study of ECM biology. It is one of a small number of ECM groups that are able to form stable cultures *in vitro* (Fries, 1977). This, in part, is why *Laccaria* species have been used to study the ecology of ECM relationships (Di Battista *et al*., 1996; Kropp & Mueller, 1999; Selosse *et al*., 2001; Smith & Read, 2008), persistence of ECM fungi in forestry and nursery studies (Molina, 1982; Henrion *et al*., 1994; Selosse *et al*., 1998), ECM fungal populations (Vincenot *et al*., 2012; Sheedy *et al*., 2015), and fungal genetics (Fries & Mueller, 1984; Wong *et al*., 1989; Gardes *et al*., 1990; Nguyen *et al*., 1992; Selosse *et al*., 1996). *Laccaria bicolor* was the first ECM fungal genome sequenced in an effort to better understand ECM evolution and genetics (Martin & Selosse, 2008; Martin *et al*., 2008).

**Figure 1:**
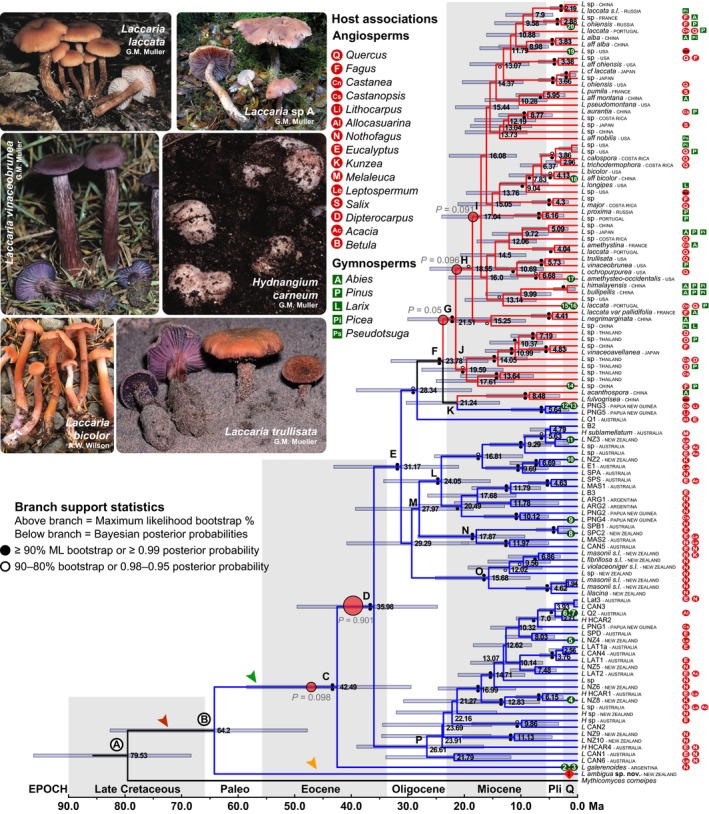
Systematic evolution, diversification and isotope profiles of *Laccaria*. Divergence dating of 116 *Laccaria* taxa. Country and host association taxon labels reflect the specimen at the terminal. Specimen IDs are provided in Supporting Information Notes S1(b). Southern and northern hemisphere taxa are represented in blue and red branches, respectively. Numbers at nodes are median estimations for time to most recent common ancestor. Gray values near red circles at nodes C, D, G, H and I are Bayesian Analysis of Macroevolutionary Mixtures marginal shift probabilities for diversification rates. The red arrow identifies the branch where *Laccaria's* ectomycorrhizal (ECM) physiology would be presumed to have evolved. The green arrow points to the branch where the *Laccaria*‐type ECM is likely to have evolved. The orange arrow points to the branch with the *L. ambigua*‐type physiology. Ma, million years ago.

*Laccaria*, including the gasteroid form *Hydnangium* (inset Fig. 1)*,* comprises at least 75 species (Kirk *et al*., 2008). Species of *Laccaria* have been reported from most regions of the world, with the exception of tropical South America (outside of *Quercus*‐dominated forest of montane Colombia) and sub‐Saharan Africa (Mueller, 1992). The genus is resolved as sister to a group of litter‐decomposing fungi, the Psathyrellaceae (Matheny *et al*., 2006). However, a recent study resolved the Hydnangiaceae as sister to a lineage that contains both saprotrophic taxa and ECM families Inocybaceae, Cortinariaceae, and Hymenogastraceae (Dentinger *et al*., 2016). Neither of these phylogenetic relationships with Hydnangiaceae are statistically supported. As a result, it is assumed that the most recent common ancestor to *Laccaria* was saprobic, which is consistent with all ECM lineages (Hibbett *et al*., 2000; Floudas *et al*., 2012; van der Heijden *et al*., 2015). This makes it possible to evaluate the origin and effect of ECM ecology on the genus. Currently, neither the age of the most recent common ancestor (MRCA) for all *Laccaria* nor the relative relationship of *Laccaria* taxa to the MRCA are known. This limits the effectiveness of comparative genomic analysis and the ability to explore hypotheses regarding the evolution of ecological roles in the genus.

Ecologically, species of *Laccaria* range from being mycorrhizal generalists to showing a strong host preference. Depending on the species, *Laccaria* taxa form ECM associations with hosts in the Pinaceae, Myrtaceae, Salicaceae, Fagaceae, Dipterocarpaceae, Nothofagaceae and a limited number of Fabaceae (Mueller, 1992). Some species have been observed to be early colonizers on the roots of young trees and are frequently found in young and old ECM forests (Deacon & Fleming, 1992; Nara *et al*., 2003). Lastly, despite *Laccaria's* ability to form stable cultures *in vitro*, there is no evidence of any species functioning as free‐living in nature.

Evaluating the evolution of *Laccaria's* ECM or saprotrophic (i.e. free‐living) capabilities requires an understanding of how species throughout the clade acquire nutrition. Natural stable isotope (^15^N and ^13^C) content can effectively identify nutritional strategies in fungi (Hobbie *et al*., 2001; Mayor *et al*., 2009). There tends to be an inverse relationship between ^13^C and ^15^N abundance, where tissues of ECM fungi have low ^13^C and high ^15^N and saprotrophic fungi have high ^13^C and low ^15^N. Understanding the variation in ^15^N and ^13^C abundance among *Laccaria* species can help to identify shifts in the nutritional physiology in relation to the evolution of the genus.

Several studies have described the diversity of *Laccaria* from various regions (Mueller, 1992; Osmundson *et al*., 2005; Vincenot *et al*., 2012; Sheedy *et al*., 2013; Wilson *et al*., 2013; Popa *et al*., 2014, 2016; Montoya *et al*., 2015). These studies are limited to relatively small geographical areas and/or datasets that do not resolve critical systematic relationships within the genus. Thus, fundamental questions regarding its true taxonomic diversity, biogeography and evolution remain. This study addresses these important gaps through extensive taxonomic and geographic sampling with multigene phylogenetic analysis to resolve systematic relationships among *Laccaria* taxa, providing the most comprehensive estimation of biodiversity in the genus to date. The resulting data have facilitated insight and further exploration into *Laccaria's* physiological and ecological evolution using a combination of molecular dating, diversification analysis and analysis of stable carbon and nitrogen isotopes.

## Materials and Methods

### DNA extraction, PCR and cycle sequencing

Protocols for DNA extraction, PCR, cloning and cycle sequencing followed those described in Wilson *et al*. (2011). The following primers were used for PCR and cycle sequencing: ITS1F (Gardes & Bruns, 1993) and ITS 4 (White *et al*., 1990) for the nuclear ribosomal internal transcribed spacer regions 1 and 2, including the 5.8S coding region (ITS); LR0R and LR5 (Vilgalys & Hester, 1990) for domains 1–3 of the nuclear ribosomal large subunit (28S); f RPB2‐5F (Liu *et al*., 1999), b RPB2‐7R, bRPB2‐7R2 and bRPB2‐7.1R (Matheny, 2005) for RNA polymerase II, subunit 2 (RPB2) regions 5–7; RPB1‐Af and RPB1‐Cr (Matheny *et al*., 2002) for RNA polymerase II, subunit 1 (RPB1), regions A–C; and EF1‐983F, EF1‐2218R, EF1‐1953R and EFcf (Rehner & Buckley, 2005) for translation elongation factor 1‐α (EF1α). Newly generated sequences were edited using Codon Code Aligner v.3.5.7 (CodonCode Corporation, Dedham, MA, USA, http://www.codoncode.com/) with generic‐level identities for sequences confirmed via Blast queries of GenBank (http://www.ncbi.nlm.nih.gov/). Nucleotide datasets were assembled using a combination of new sequences and those derived from GenBank. Datasets were aligned using Muscle v.3.8.31 (Edgar, 2004) with default settings, followed by manual alignment using Mesquite v.2.75 (Maddison & Maddison, 2015).

### Phylogenetic analysis

Three molecular datasets were created for analysis: the *Laccaria* Systematics dataset was assembled using ITS, 28S, RPB2 and EF1α sequence data from a comprehensive sampling of *Laccaria* from all over the world in order to delimit phylogenetic species for the following datasets; the Global *Laccaria* dataset was assembled for molecular dating of *Laccaria* using ITS, 28S, RPB2 and EF1α sequence data, and phylogenetic species from analysis of the *Laccaria* Systematics dataset; and the Agaricomycetideae dataset was assembled using nuclear ribosomal small subunit (18S), ITS, 28S, RPB1, RPB2 and EF1α sequence data and a broad sampling of taxa from within the Agaricomycetideae as a preliminary step to calibrate nodes for molecular dating of the Global *Laccaria* dataset. The taxonomic compositions of these datasets are provided in Supporting Information Notes S1 and S2.

Throughout this study, ‘*Laccaria*’ will be discussed as inclusive of *Hydnangium* (i.e. Hydnangiaceae). All analyses were implemented on the CIPRES web portal (http://www.phylo.org/portal2/) (Miller *et al*., 2009). Maximum likelihood bootstrap (MLB) analyses were performed using RAxML v.2.2.3 (Stamatakis, 2006). A total of 1000 bootstrap replicates were performed under the GTR + I + G model of evolution. Bootstrap support from maximum likelihood analysis ≥ 80% is reported on the branches of phylogenies. Maximum likelihood bootstrap support ≥ 90% is considered as ‘strong’ support. Bayesian Metropolis‐coupled Markov chain Monte Carlo (MCMC) analyses were performed using the GTR + I + G model of evolution in MrBayes v.3.1.2 (Ronquist & Huelsenbeck, 2003). The analyses used four chains and sampling every 100^th^ tree for 10 million generations. The burn‐in to be removed was determined using LogCombiner v.1.8.0 (Drummond & Rambaut, 2007) either by taking the first 10% of the iterations, or, if convergence around a stable average likelihood involved > 10% of the iterations, by simply removing this proportion of trees from the analysis. Bayesian posterior probabilities (PPs) ≥ 0.95 are reported with MLB support on the branches of the phylogenies, with PP ≥ 0.98 considered ‘strong’ support.

### Divergence time estimation

The two‐step calibration procedure described by Renner (2005) was used to estimate node ages using Beast v.1.8.0 (Drummond & Rambaut, 2007). This method has been employed for fungi in several cases (Skrede *et al*., 2011; Ryberg & Matheny, 2012; Wilson *et al*., 2012). We used Beauti v.1.8.0 to create XML files for datasets representing Agaricomycetidae and Global *Laccaria*. Individual nexus files were created for the 18S, ITS, 28S, RPB1, RPB2 and EF1α gene regions. The start of the RPB1, RPB2 and EF1α nexus files was adjusted so the first nucleotide in the matrix reflects the first nucleotide of the codon reading frame. Introns for these gene regions were also removed. All nexus files were then uploaded into Beauti and the following analytical settings were implemented: GTR model, uncorrelated relaxed clock with lognormal rate distribution; estimating separate rates for genes ITS, 18S, 28S, RPB1, RPB2 and EF1α; a two‐codon partition ((1 + 2), 3) was used for RPB1, RPB2 and EF1α; the Tree Prior was set to Speciation: Birth‐Death Process; and each analysis was run with 10 million generations, sampling every 1000^th^ tree. Analysis of each dataset was run at least three times to establish independence between each Bayesian search. The burn‐in was removed under the same conditions as the Bayesian analyses described earlier. Means and 95% highest posterior density (HPD) for nodes of interest were examined from Beast logfiles using Tracer v.1.5 (Drummond & Rambaut, 2007). A summary tree was produced using LogCombiner v.1.8.0 to combine the posterior trees from all Beast analyses into a single treefile, followed by TreeAnnotator v.1.8.0 (Drummond & Rambaut, 2007) to summarize the trees into a single summary tree.

The Agaricomycetidae dataset consisted of the following taxonomic groupings defined in Beauti: Suillineae, marasmioid fungi, all *Laccaria*,* Laccaria* minus *L. ambigua* (see later) and northern hemisphere *Laccaria*. Two nodes were calibrated using fossil data: the marasmioid fungi (*Marasmius rotula* and *Mycena amabillisima*) based on a 90 Ma fossil *Archaeomarasmius legetti* from mid‐Cretaceous amber (Hibbett *et al*., 1997); and the Suillineae (*Suillus pictus* and *Gomphus roseus*) using a 50 Ma permineralized suilloid ECM fossil associated with pine roots (LePage *et al*., 1997). These two groups were the only ones constrained to be monophyletic. This allowed the Bayesian search in Beast to freely identify the outgroup for *Laccaria* based on the molecular data. Details of parameter values used in calibrating the time to MRCA are given in Notes S3.

In the Global *Laccaria* dataset, 11 taxonomic groups were defined to evaluate time to MRCAs. These include the calibrated nodes *Laccaria* plus the outgroup *Mythicomyces corneipes* (= node A), and all *Laccaria* (= node B). Calibrations were set using a lognormal distribution. The nested, two‐step calibration method used in this analysis has a tendency for Beast to produce younger age estimations than initially provided by the priors. To counteract this, the priors for mean age and standard deviation (SD) of node A were set according to the older date for this node provided by Floudas *et al*. (2012). To approximate the age and HPD for internal node B of *Laccaria,* the time to MRCA estimate from the Agaricomycetideae dataset was used to establish the offset, mean and SD priors for analysis in Beast (Notes S3).

### Sister clade analysis

To better understand the potential of *Laccaria's* direct ancestor being saprobic, a Shimodaira–Hasegawa test (Shimodaira & Hasegawa, 1999) was performed on the Agaricomycetideae dataset to compare potential sister‐clade relationships with other possible ECM lineages. Any nonrejected sister relationship between *Laccaria* and another ECM lineage signifies that an ECM ancestor for *Laccaria* is possible.

The Shimodaira–Hasegawa test was performed using the SH.test() function from the package phangorn (Schliep, 2011) implemented in R (http://www.r-project.org/). An unconstrained phylogenetic analysis of the Agaricomycetideae dataset was compared with constraints of other ECM lineages, such as *Laccaria* + *Inocybe*,* Laccaria* + *Cortinariaceae* and *Laccaria* +*Hebeloma*.

### Diversification analysis

Detecting the location of diversification rate shifts in *Laccaria* was performed using the C++ program Bayesian Analysis of Macroevolutionary Mixtures (BAMM) (Rabosky, 2014). BAMM analysis used the Global *Laccaria* phylogeny generated in Beast, and was performed both with and without the outgroup *M. corneipes*. The prior control block was produced using the setBAMMpriors function from the R package BAMMtools (Rabosky *et al*., 2014). The remaining settings were as follows: numberOfGenerations = 1000 0000; mcmcWriteFreq = 10 000; eventDataWriteFreq = 5000; printFreq = 1000; acceptanceResetFreq = 10 000. Analysis of BAMM results included generating the mean phylorate plot, producing the marginal shift probabilities for rate shifts among branches, and evaluation of 95% credible set of macroevolutionary rate configurations (AKA credible shift sets) using BAMMtools.

Binary state speciation and extinction analysis (BiSSE) (Maddison *et al*., 2007) was used to estimate rates of speciation (*λ*), extinction (*μ*) and state transformation (q01 and q10) associated with southern hemisphere (state 0) and northern hemisphere (state 1) *Laccaria*. The analysis was implemented in R using the package Diversitree v.0.4‐3 (FitzJohn *et al*., 2009). LogCombiner v.1.8.0 was used to randomly sample 1000 ultrametric trees from the Beast time to MRCA analysis of the global *Laccaria* dataset for the BiSSE analysis. A MCMC method was used for sampling rate parameters on each tree, with 100 iterations performed per tree discarding the first 25% of the iterations as part of the burn‐in. This produced a total 75 000 iterations from which to calculate the means and the 95% posterior densities for distributions of state‐associated speciation and extinction parameters.

Character state diversification rates (*r*) are defined as the difference between the speciation and extinction rates for a particular state (i.e. southern hemisphere *Laccaria* diversification rate = *r*0 = *λ*0 − *μ*0). The relative diversification rate (*r*_rel_) between two states was calculated by dividing the diversification rate of southern hemisphere *Laccaria* by the diversification rate of northern hemisphere *Laccaria* (i.e. *r*_rel_ =* r*0/*r*1).

A likelihood‐ratio test was performed to compare northern and southern hemisphere speciation and diversification rates. Using the 1000 previously sampled trees, Diversitree's find.mle function was used to produce maximum likelihood estimations of diversification rates on each phylogeny under three models: one unconstrained model (no constraints on any rate parameter); a speciation null model with speciation rates constrained to be equal (*λ*0 = *λ*1); and a diversification null model with both speciation and extinction rates constrained to be equal (*λ*0 = *λ*1 and *μ*0 = *μ*1). Comparison of likelihood scores from the unconstrained model against those of the speciation and diversification null models was performed using the ANOVA function in R to obtain *χ* ^2^ and *P* scores.

### Isotopic analysis and sample preparation

Isotopic analyses were used to establish the nutritional and metabolic profile of *L. ambigua* (as provided by specimen PDD89696). This was done by comparing the stable carbon (δ^13^C) and nitrogen (δ^15^N) isotopes of specimen PDD89696 with other *Laccaria* species and other fungi representing various fungal nutritional modes (Hobbie *et al*., 2001). A total of 37 specimens were analyzed, including 20 *Laccaria* specimens (including *L*. *ambigua*), eight ECM species, seven saprotrophic species, and two of unknown ecology. From each sample, a minimum of 10 mg basidiome tissue, dried at 50°C, was ground to a fine powder using a mortar and pestle. From this, 3 mg was analyzed for δ^13^C and δ^15^N abundance at the University of New Hampshire Stable Isotope Laboratory by continuous flow with a Costech ECS4010 elemental analyzer (Costech Analytical Technologies Inc., Valencia, CA, USA) coupled with a Geo Vision isotope ratio mass spectrometer (Elementar Americas, Mt. Laurel, NJ, USA). Stable isotope abundances are reported as: δ^15^N or δ^13^C (‰) = (*R*_sample_/*R*_standard_ −1) × 1000, where *R* is the ratio ^13^C/^12^C or ^15^N/^14^N. All δ^13^C and δ^15^N values were normalized on Vienna‐PeeDee Belemnite (δ^13^C) and atmospheric nitrogen (δ^15^N) reference scales with laboratory working standards of NIST 1515 (apple leaves), and tuna muscle, as well as an internal *Boletus* standard. A meta‐analysis of the data generated for this study and isotopic profiles of global ECM fungi, saprotrophic fungi, and other *Laccaria* taxa was performed using the data aggregated by Mayor *et al*. (2009).

## Results

### Dataset composition

The 237 sample *Laccaria* Systematics dataset was analyzed under maximum likelihood and Bayesian methods. A combination of moderate to strong support for clades, as well as individual samples with sufficient branch length (Notes S1) was used to select the 116 phylogenetic species of *Laccaria* to make the Global *Laccaria* dataset. One species in the dataset is described as a new *Laccaria* species, *L. ambigua,* in this study (Box 1). Information on dataset length and composition can be found in Notes S2(c). The southern hemisphere *Laccaria* taxa were labeled according to Sheedy *et al*. (2013). All datasets are deposited in TreeBase (http://purl.org/phylo/treebase/phylows/study/TB2:S18946).

Box 1 **Species description of *Laccaria ambigua***

***Laccaria ambigua*** K. Hosaka, A. W. Wilson, & G. M. Mueller sp. nov.

Box 1 figure, panels a–d.

Mycobank ID: MB 818267

Diagnosis: pileus up to 10 mm diameter, plane to uplifted, not striate, hygrophanous, orange brown. Lamellae thick and waxy, vinaceous brown. Stipe up to 17 mm × 4 mm, equal, glabrous, concolorous with pileus. Basidiospores globose, 9–10 μm, finely echinulate. Basidia tetrasterigmate. Growing in forest dominated by nonectotrophic trees.

Type: New Zealand, North Island, Coromandel Peninsula, near town of Coromandel, Waiau Kauri Grove Walk, 36°49′34.94″S 175°32′47.79″E, 9‐V‐06, PDD89696 (KH‐NZ06‐082) (holotype; PDD).

Etymology: name refers to the ambiguous status of its plant associate and mycorrhizal ecology.

Pileus: 8–10 mm diameter, plane to uplifted, slightly depressed, subglabrous, not striate, hygrophanous, orange brown fading to ochraceous buff. Lamellae: thick, waxy appearing, entire, vinaceous brown. Stipe: 15–17 × 4 mm, equal, glabrous, concolorous with pileus.

Basidiospores: (*n* = 20) (8.7‐)9–9.6(−10.3) × (8.5‐)9–9.6(−10.3) μm, mean = 9.4 × 9.35 μm, *Q* = 1–1.02, globose, finely echinulate 1.5–2 × < 1 μm. Basidia: (*n* = 5) 32–40 × 9.5–11 μm, tetrasterigmate, sterigmata 5–6 μm long. Hyphae with clamp connections.

Habitat and distribution: collected from the Coromandel Peninsula on the North Island of New Zealand, trailside along mossy bank where no obvious ectomycorrhizal trees were observed near basidocarps. There are Kauri (*Agathis australis*, Araucariaceae) trees, tree ferns and a few other nonectomycorrhizal trees present, but no Nothofagaceae or Myrtaceae (*Leptospermum* and *Kunzea*).

Sequence data: nuclear ribosomal internal transcribed spacer region (ITS) = KU685725, nuclear ribosomal large subunit (28S) = KU685876, RNA polymerase 2, second subunit (RPB2) = KU686018, elongation factor 1 alpha (EF1a) = KU686132.

Notes: *Laccaria ambigua* is currently known from a single specimen consisting of two basidiocarps. Morphologically it is similar to *L. laccata* sensu lato, but is differentiated by its thicker lamellae and habitat. It is unambiguously differentiated and resolved as basal to the rest of the genus with sequence data (Box 1 figure ).

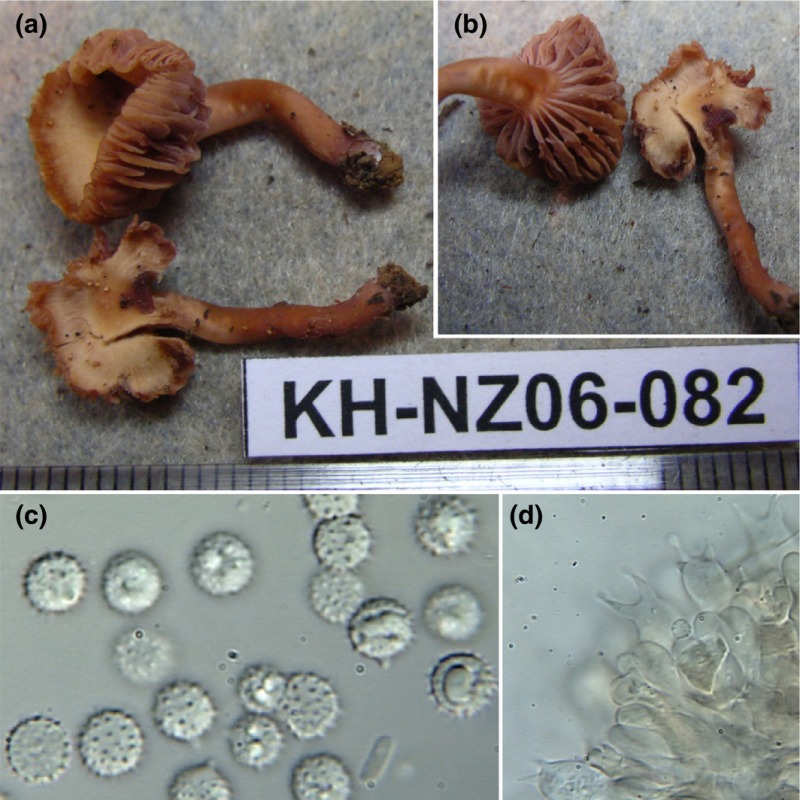

Box 1 figure Morphology of *Laccaria ambigua* PDD89696 from New Zealand. (a, b) Profiles of *Laccaria ambigua* basidiomes; (c) basidiospores (×1000); (d) basidia (×400).

### Molecular dating

All ages are represented in units of millions of yr ago (Ma). Ages from the Agaricomycetideae dataset analysis overlap in HPD estimates from Floudas *et al*. (2012). Ages from the Agaricomycetideae analysis representing the most recent common ancestor for *Laccaria* + the Psathyrellaceae (Notes S4a: node A, median age of 84.39 Ma, HPD 65.16–112.51 Ma), and for *Laccaria* (node B, 56.91 Ma, HPD 39.14–79.82 Ma) were used for two‐step calibration of the Global *Laccaria* dataset (Notes S4b).

Results for the molecular dating analysis of the Global *Laccaria* dataset are presented in Fig. 1a. The most recent common ancestor at the split between *Laccaria* and *M. corneipes* (node A) has a median age of 79.53 Ma (HPD 68.32–96.14). This is slightly younger but still consistent with the results of the Agaricomycetidae dataset (Notes S4; median age 84.39 Ma). The estimated median age of node B is 64.20 Ma (HPD 47.70–82.62), older than that of the Agaricomycetidae dataset (56.90 Ma, HPD 39.14–79.82), which seems to be a response to accommodating all the additional taxa contained within this group.

Nodes labeled A–P on the Global *Laccaria* phylogeny identify important and well‐supported lineages in the evolution of *Laccaria* (Fig. 1). Phylogenetic analyses demonstrate a strong biogeographic pattern, indicating that basal lineages are solely distributed in the southern hemisphere, that is, Australasia with sparse representation in temperate South America. Node F represents the MRCA for all northern hemisphere‐distributed taxa. Nodes C–I consist of well‐supported branches along the backbone of *Laccaria*, with nodes G, H and I representing branches where minor diversification rate shifts are detected for northern hemisphere *Laccaria*. Letters J and on represent clades consisting of more than three taxa that have notable geographic distribution patterns: clade J is a predominantly Southeast Asian clade; clade K contains two distinct lineages, one representing *Laccaria* from China, and the other from Papua New Guinea; clade M contains clades L and N; clade L represents a wide geographic range of *Laccaria* from Argentina, Australia, New Zealand and Papua New Guinea; clade N represents species from both Australia and New Zealand; clade O contains *Laccaria* species only found on New Zealand's South Island; and P represents Australasian *Laccaria* and *Hydnangium* species, but is an unsupported clade that was consistently resolved in all phylogenetic analyses.

### Sister clade analysis

The results indicate that constraints between *Laccaria* + Inocybaceae and *Laccaria* + *Hebeloma* produced a significantly worse likelihood result when compared with the unconstrained tree (Table 1), and these relationships are rejected (*P*‐value < 0.05). The *Laccaria* + Cortinariaceae relationship produced a worse likelihood score than the unconstrained tree, but was not rejected in the analysis.

**Table 1:** Results of Shimodaira–Hasegawa test of *Laccaria's* potential sister relationship to other ectomycorrhizal (ECM) lineages

Tree	log_e_ L	Diff log_e_ L	*P*‐value
Unconstrained	−57 047.13	0.00	0.762
+Cortinariaceae	−57 056.39	9.26	0.693
+Inocybaceae	−58 823.74	1776.60	0.000
*+Hebeloma*	−58 835.71	1788.57	0.000

The Beast analysis of the Agaricomycetidae dataset resolved *Laccaria* with the Psathyrellaceae, which was also observed in Matheny *et al*. (2006) and was given high Bayesian (1.0 BPP) but low MLB support (61%) in this study (Notes S3). By comparison, the Cortinariaceae was sister to the crown Agaricineae (Inocybaceae, Hydnangiaceae, Strophariaceae, etc.), with a 1.0 BPP support and 81% MLB support.

### Diversification analysis

Analysis of diversification in *Laccaria* using BAMM, regardless of the presence of *M. corneipes* as the outgroup, identified a major diversification rate shift occurring at the base of the tree leading to node D (Fig. 1). This particular shift has the largest marginal shift probability (= the probability that a rate shift occurred along that branch) of all shifts detected (*P* = 0.901). Credible shift set results from BAMM identify the most probable sets of branches that best explain the data. In this study, node D was included in four of the seven credible shift sets recovered (Notes S5b). There is a minor shift on the branch immediately ancestral, leading to node D, in three of the seven total credible shift sets. This branch has a marginal shift probability of 0.098. One or the other of these two shifts in diversification rate are featured in each of the seven credible shift sets, but never together. Minor shifts at nodes G, H and I were recovered in five of the credible shift sets. These nodes had marginal shift probabilities of 0.050, 0.096 and 0.091, respectively, and are associated with *Laccaria's* dispersal throughout the northern hemisphere.

Difference in rates of speciation and diversification between northern and southern hemisphere *Laccaria*, as evaluated using BiSSE, are presented as histograms in Notes S6. The 95% HPDs for distributions of speciation and diversification rate comparisons overlap between the two hemispheres, but northern hemisphere *Laccaria* is estimated to be diversifying at a rate of more than twice that of the southern hemisphere (*r*_rel_ = 0.485).

The likelihood ratio test between northern and southern hemisphere speciation rates produced a mean *P*‐score comparison of 0.075, which distinguishes the unconstrained model from the null model (where *λ*_northern_ = *λ*_southern_), when assuming a *P* < 0.1 cutoff. The difference in likelihood ratio scores between the diversification unconstrained and null models is even more distinct, with a mean *P*‐score of 0.014, demonstrating that northern hemisphere *Laccaria* are diversifying at a faster rate than southern hemisphere *Laccaria* (Notes S6).

### Isotopic analysis of *L. ambigua*

**Figure 2:**
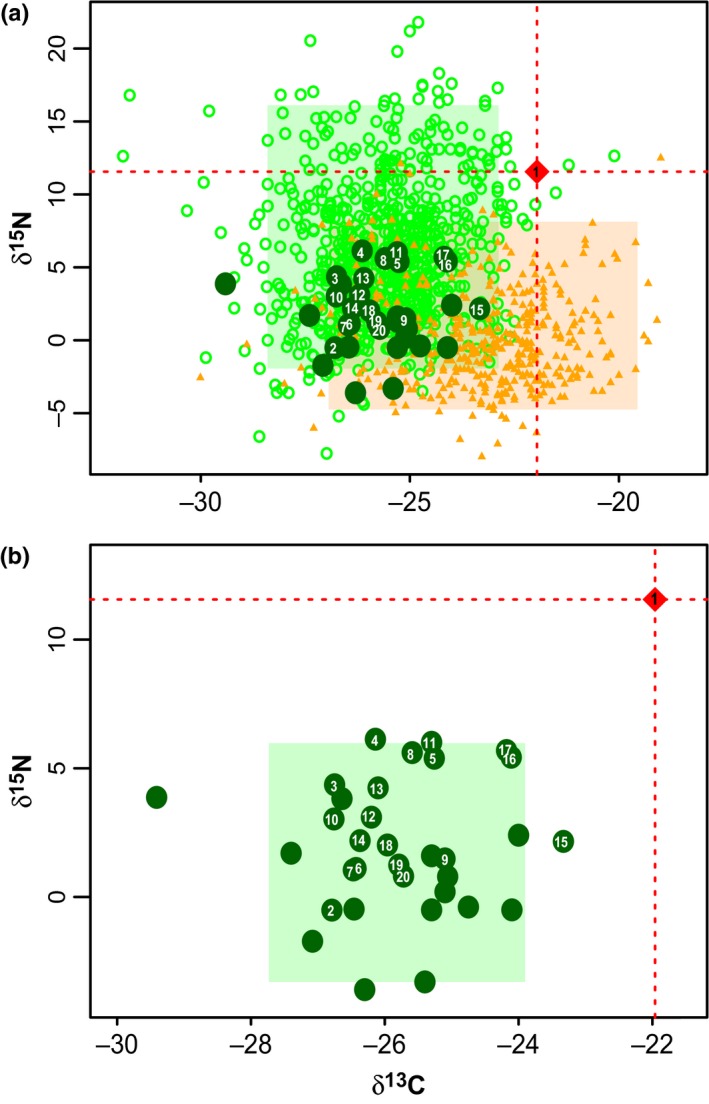
Stable δ^13^C and δ^15^N isotope profiles of *Laccaria ambigua* (red diamond) are compared with known ectomycorrhizal (ECM; open green circles), saprotrophic fungi (orange triangles) and other *Laccaria* taxa (closed dark green circles). Numbers in closed dark green circles refer to known *Laccaria* taxa identified by the terminal branches of Fig. 1. Shaded rectangles represent 95% highest posterior density of δ^13^C and δ^15^N values for ECM fungi (green, in a), saprotrophic fungi (orange, a), and *Laccaria* (green, in b).

In Fig. 2, the δ^13^C signature for *L. ambigua* (–21.96) is outside the 95% CIs for ECM fungi (CI = −28.44 to −22.83) but within the range for saprotrophs (CI = −26.98 to −19.53). However, δ^15^N (11.56) is within the 95% CI for ECM fungi (CI = −2.05 to −16.27), but outside that of saprotrophic fungi (CI = −4.84 to −8.22). When compared with other *Laccaria* taxa alone (Fig. 1 b2), *L. ambigua* is outside the 95% CI for both δ^13^C (CI = −28.25 to −24.06) and δ^15^N (CI = −3.43 to 6.06). Sporocarp δ^13^C and δ^15^N values generated for this study are provided in Notes S7.

## Discussion

Phylogenetic analysis of the *Laccaria* Systematics dataset identified 116 phylogenetic species of *Laccaria* (Notes S1). These were used for assembling the Global *Laccaria* dataset (Fig. 1). This represents 50% more taxa than previously reported for the genus (Kirk *et al*., 2008). Apart from the new species, *L. ambigua* (Box 1), identification and description of *Laccaria* taxa are a work in progress. The *Laccaria* Systematics phylogeny (Notes S1) uses branch labels that identify morphospecies, which are often field identifications. The phylogenetic species concept employed here, and in the work of Sheedy *et al*. (2013), reveals how inaccurate the morphological species concept is for identifying *Laccaria* species with broad geographic ranges (e.g. *L. ohiensis*). Recently, new species of *Laccaria* in the northern hemisphere have been described, or suggested based on molecular analysis (Vincenot *et al*., 2012; Popa *et al*., 2014, 2016; Montoya *et al*., 2015). An analysis of a *Laccaria* ITS sequence dataset consisting of > 800 sequences (data not shown) indicates that two of the five new species from these studies (*L. aurantia* and *L. fulvogrisea*) are represented in the *Laccaria* Systematics dataset. The three species that are absent (*L. roseoalbescens, L. stellate* and *L. yunnanensis*) fall within the northern hemisphere clade of the ITS tree, and thus their exclusion does not upset the broader phylogeographic and systematic evaluation of *Laccaria* evolution in this study. Results of ongoing collaborative work on southern hemisphere *Laccaria* suggest there are more cryptic taxa to be identified there (Sheedy *et al*., 2013). While further fieldwork is expected to uncover additional *Laccaria* species, this study represents the most complete sampling of the genus ever assembled for systematic analysis and covers the group's full geographic range. Thus, the datasets used in this study provide an effective framework in which to evaluate the evolutionary history of *Laccaria*.

### Systematic evolution and biogeography of *Laccaria*

The most recent common ancestor to all *Laccaria* originated *c*. 64 Ma in Australasia (Fig. 1, node B). The supercontinent Gondwana was broken up by this time. As a result, the current distribution of *Laccaria* is best described as the result of dispersal rather than vicariance. Long‐distance dispersal between isolated populations of extant fungi is understood to be rare (Peay *et al*., 2010). However, it is apparently a successful strategy over evolutionary time frames because it is the most probable mechanism for explaining extant distributions of related Agaricomycetes separated by geographic barriers (Martín *et al*., 2002; Hosaka *et al*., 2008; Geml *et al*., 2012; Sheedy *et al*., 2015).

The southern hemisphere ECM hosts of extant *Laccaria* include the Nothofagaceae, and members of the Myrtaceae and Fabaceae. The Nothofagaceae have been used for understanding southern hemisphere biogeography, and molecular dating indicates that this family owes its distribution to long‐distance dispersal (Knapp *et al*., 2005). A similar story can be told for the Myrtaceae as this family (*c*. 70 Ma) also postdates the breakup of Gondwana (Sytsma *et al*., 2004). Ancestral species from the Nothofagaceae and Myrtaceae taxa were probably ECM associates during the early evolution of *Laccaria*. The dispersal of these hosts would facilitate dispersal of *Laccaria* taxa, helping to explain its Australasian distribution. Ancestral state reconstruction of the ECM hosts for *Laccaria* would be ideal to explore the significance of host associations, but current identification of hosts is subjective, making accurate reconstruction of host associations unlikely (Wilson *et al*., 2012). Further evaluation of *Laccaria's* southern hemisphere phylogeography along with molecular analysis of ECM roots is the next step in documenting possible coevolutionary patterns in ECM host associations.

*Laccaria’*s dispersal to the northern hemisphere probably began in the late Oligocene to early Miocene. Starting in Papua New Guinea, the genus dispersed through Southeast Asia before eventually reaching Laurasia's diverse mesophytic forests. While dispersal from Papua New Guinea into Southeast Asia may have been facilitated by associations with *Nothofagus* (Morley, 2001), dispersal into the Northern Hemisphere was most probably made possible by host switching to the Fagaceae. Associations with the Fagaceae are observed as far south as Papua New Guinea, where *Laccaria* in clades K, L and P (Fig. 1) were collected under *Castinopsis*, as well as *Lithocarpus* in clade K. The geographic history of the Fagaceae describes a late Eocene, early Oligocene origin in Southeast Asia with subsequent dispersal to Europe and North America (Manos & Stanford, 2001). *Laccaria's* association with ECM hosts in the Fagaceae would facilitate its dispersal north throughout Laurasia, followed by additional host jumps to the Salicaceae and Pinaceae. This potentially provided opportunities for dispersal of *Laccaria* into new northern hemisphere niches in which it could diversify.

### Diversification rate shifts in association with the evolution of the ECM ecology in *Laccaria*

The results of this study and the conclusions drawn from them are based in part on the assumption that *Laccaria* shares a recent common ancestor with the Psathyrellaceae (Fig. 1; Notes S3, node A). The key assumption is recognizing the ancestor to *Laccaria* as saprotrophic. Below we discuss the individual results of this study and how the accumulated evidence points to a unique origin of the *Laccaria* ECM ecology.

As a sister relationship with the Psatherellaceae reflects a saprotrophic state for the ancestor between the Psathyrellaceae and *Laccaria* (Fig. 1, node A), it then follows that the evolution of the ECM ecology occurred in the lineage leading to node B (red arrow). However, this leads to the question of why the most significant diversification rate shift detected in *Laccaria* was identified along the branch leading to node D (Fig. 1). One possible explanation is that node B does not identify the MRCA to ECM within *Laccaria*, but that this ecology evolved later. Assuming the transition to ECM occurred after node B, and along the branch leading to node C, then the proximity to the diversification rate shifts on the *Laccaria* phylogeny suggests that these two events are correlated, despite having only a sample size of one.

The diversification of *Laccaria* at node B leads to two lineages. One represents > 99% of all *Laccaria*, the other is represented by a single species, *L. ambigua* (Box 1). This taxon represents an enigmatic species of *Laccaria*, which is currently the only known extant member of this lineage, and consists of a single collection (PDD89696). This was collected in an *Agathis australis* (also known as ‘Kauri’) grove *c*. 30 m from the nearest observed ectotrophic host (*Leptospermum*; Myrtaceae). *L. ambigua* is intriguing because it has the characteristic *Laccaria* macro‐ and micromorphology (Box 1) and is well supported as within the genus, but derived from the ancestor at node B, in all analyses. The lack of nearby New Zealand ECM hosts (i.e. *Leptospermum*,* Kunzea*,* Fuscospora*,* Lophozonia*) instigated questions about this species’ free‐living capacity. The elevated δ^15^N profile of collection PDD89696 suggests that *L. ambigua* is potentially ECM‐like in its ecology. However, this assessment is contradicted by the sample's elevated δ^13^C profile, which is consistent with the predominant signature of a saprotrophic fungus (Hobbie *et al*., 2001; Mayor *et al*., 2009). Typically, isotopic measurements between sporocarps are comparable within 50 × 50 m quadrats to ensure the fungi are drawing from common C and N sources. As a result, several other sporocarps from Kauri Grove – representing fungi of saprotrophic and unknown ecology – were sampled for isotopic analysis (Notes S7). Unfortunately no other *Laccaria* specimens were found in the grove. Regardless, *Laccaria* taxa from nearby on the Coromandel Peninsula, elsewhere on New Zealand's North Island, and across both northern and southern hemispheres were included in the analysis. These represent the systematic diversity of *Laccaria* as well as a variety of C and N sources. The isotopic signatures of these specimens form a cluster in the isotopic profiles of Fig. 2. The isotopic profile of the collection representing *L. ambigua* (PDD89696) falls well outside of the *Laccaria* cluster. Unless the area from which collection PDD89696 was collected represents a unique source of C and N – which should show anomalies in the isotope profiles of other Kauri Grove sporocarps – its unique δ^15^N vs δ^13^C profile (Fig. 2) is most probably a result of an atypical *Laccaria* physiology in relation to the acquisition of carbon and nitrogen.

Whether the distinct physiology of *L. ambigua* is indicative of a saprotrophic or facultative ECM ecology is unclear. More collections of *L. ambigua* are needed in order to study its metabolism and genomics. This will help to establish the nature of its ecological activity and also further the role of *Laccaria* as a model for understanding ECM biology. Without additional evidence, this study will regard *L. ambigua* as saprotrophic in its nutritional role. Under this designation, the phylogenetic and isotopic analyses suggest that a physiological innovation in *Laccaria* occurred after node B (green arrow, Fig. 1) that may explain the large shift in diversification rate leading to node D.

The hypothesis that a novel, *Laccaria*‐like ECM physiology developed within the genus depends on the ancestor to all *Laccaria* as being something else. Currently it is easiest to presume a saprotrophic ancestor. However, a potential sister relationship with another ECM lineage would put this presumption under scrutiny. The Shimodaira–Hasegawa test was able to rule out all tested ECM sister relationships, except with the Cortinariaceae (Table 1). However, a sister relationship between *Laccaria* and the Cortinariaceae does not necessarily mean that their ancestor at node A was ECM (Fig. 1). A common ECM origin between these two groups would require either a reversal to a putative free‐living ecology (i.e. saprotrophy) or switch to a unique biotrophic physiology in the lineage represented by *L. ambigua* (orange arrow). Reversals from ECM biotrophy to saprotrophy have been scrutinized and deemed improbable (Bruns & Shefferson, 2004), and analysis of a comprehensive Agaricales dataset found no evidence of unambiguous reversals of the ECM ecology (Matheny *et al*., 2006). In the genus *Amanita*, the switch from saprotrophy to ECM biotrophy resulted in the loss of the ability to break down cellulose (Wolfe *et al*., 2012). This makes the reversal from the ECM ecology to saprotrophy unlikely, helping to explain why such reversals have not been observed in ECM lineages (van der Heijden *et al*., 2015). Lastly, a common origin of the ECM ecology between *Laccaria* and the Cortinariaceae would argue for homology between their genomes, which is not the case (Kohler *et al*., 2015). So the evidence suggests that even in the event of a sister relationship between *Laccaria* and the Cortinariaceae, each group adopted the ECM ecology independently of one another, *de novo*, from a saprotrophic ancestor at node A (Fig. 1). This means that the development of the *Laccaria*‐type ECM, shortly after node B (green arrow Fig. 1), coincides with a significant shift in diversification rate in the evolution of *Laccaria*.

Precedent for a *Laccaria*‐type ECM physiology is observed in a recent study of ECM genomics. A comparison of the presence and sequence similarity of up‐regulated genes in *L. bicolor* demonstrate a significant homology with the genome of *L. amethystina* (Kohler *et al*., 2015). By contrast, the genomic profiles of these two *Laccaria* species are as distinct from other ECM genera (e.g. *Amanita*,* Cortinarius*,* Hebeloma*,* Paxillus*,* Piloderma*,* Pisolithus*,* Scleroderma* and *Suillus*) as these genera are from each other. These differences among ECM fungal lineages will have different natural selective effects upon these lineages. One such effect is observed as differences in diversification rates among the lineages (Ryberg & Matheny, 2012). In *Laccaria*, the provided phylogenetic and isotopic data support the hypothesis that the early diversification in the genus was shaped by the evolution of a distinct ECM ecology.

Further evaluation of diversification detected smaller diversification rate shifts along the backbone of northern hemisphere *Laccaria*. These shifts are located at nodes G, H and I in the phylogeny (Fig. 1). The elevated diversification rate of northern hemisphere *Laccaria* indicated by the BiSSE analysis (Notes S6) is largely attributed to the availability of new niches into which *Laccaria* were able to radiate and diversify. Such observations of a species‐area effect is often used to explain the expansion of ECM communities and lineages found in temperate forests (Tedersoo *et al*., 2012). However, the enhanced rate of diversification and broad distribution of *Laccaria* species make it difficult to identify any clear phylogeographic patterns within the northern hemisphere. One hypothesis regarding the dispersal into the northern hemisphere suggests that as *Laccaria* dispersed from Australasia to Southeast Asia, an early association with the Fagaceae aided in its transition to north temperate forests and provided opportunities for the genus to associate with new hosts, habitats and the niches available in the northern hemisphere. While further evaluation of *Laccaria's* northern hemisphere biogeographic history is necessary, it will require finer‐scale phylogenetic or even population genetic analysis to sift through the evolutionary noise and deconstruct the genus's history in this part of the world.

Using BiSSE to test hypotheses regarding state‐specific diversification rates has come under criticism for being prone to introduced bias, resulting in errors in estimating diversification rates. One such source could stem from improper character state interpretation (Maddison & FitzJohn, 2014). However, this is not likely because interpretation of *Laccaria* species as either from the northern or southern hemisphere is not ambiguous. Type I errors could occur if northern hemisphere *Laccaria* is overrepresented in the analysis as the results suggest it has a higher rate of diversification (Davis *et al*., 2013). However, this is not the case and it is more likely that potential undersampling is greater in the northern than in southern hemisphere given the potential for – and reality of – unsampled cryptic species. Lastly, what have been interpreted as type II errors in BiSSE are unlikely because character states are dispersed consistently within a single northern hemisphere clade and among a clear southern hemisphere grade. This alleviates the issues that BiSSE has with estimating the rate of character state changes in the phylogeny (Rabosky & Goldberg, 2015).

## Conclusions

The current study provides a thorough evaluation of *Laccaria's* systematic diversity and is one of the most taxonomically comprehensive studies of any group of Agaricomycetes. Although the results have identified > 50% more *Laccaria* species, the cryptic nature of fungi makes it likely that undescribed taxa remain to be discovered. Regardless, the evaluation of evolutionary events in *Laccaria* suggests that the genus is defined by diversification shifts and dispersal events associated with its ECM ecology and dispersal throughout the northern hemisphere. This is seen in stable isotope analyses that show a difference in nutritional physiology between the primary *Laccaria* lineage and its sister lineage currently represented by a single extant taxon. Given *Laccaria's* history as a model of ECM ecology in fungi, these results further establish the importance of the genus in the exploration of ECM symbiosis.

## Author contributions

A.W.W. produced sequence data, conceived of and performed analyses, and wrote the study. G.M.M. and K.H. collected the majority of samples used. K.H. produced the majority of sequence data and performed preliminary analysis. G.M.M. conceived of the study and contributed to its writing.

## Supporting information

Please note: Wiley Blackwell are not responsible for the content or functionality of any Supporting Information supplied by the authors. Any queries (other than missing material) should be directed to the *New Phytologist* Central Office.

**Notes S1** *Laccaria* Systematics and Global *Laccaria* phylogenies with collection ID numbers.
**Notes S2** Specimen information and GenBank sequence IDs.
**Notes S3** Agaricomycetideae phylogeny and calibration priors for time to MRCA analysis.
**Notes S4** Agaricomycetideae and Global *Laccaria* time to MRCA results.
**Notes S5** BAMM results.
**Notes S6** BiSSE results.
**Notes S7** Isotope data and specimen information.
