# Supplementary material for: Evolution of ectomycorrhizas as a driver of diversification and biogeographic patterns in the model mycorrhizal mushroom genus Laccaria
Source: New Phytol. 2016 Nov 7;213(4):1862–73. doi: 10.1111/nph.14270 (PMC5324586; doi:10.1111/nph.14270)
Supplement: Supplementary file 1 — Notes S1 Laccaria Systematics and Global Laccaria phylogenies with collection ID numbers. Notes S2 Specimen information and GenBank sequence IDs. Notes S3 Agaricomycetideae phylogeny and calibration priors for time to MRCA analysis. Notes S4 Agaricomycetideae and Global Laccaria time to MRCA results. Notes S5 BAMM results. Notes S6 BiSSE results. Notes S7 Isotope data and specimen information. [file NPH-213-1862-s001.pdf]

New Phytologist Supporting Information (Notes)

Article title: Evolution of ectomycorrhizas as a driver of diversification and biogeographic patterns in the model mycorrhizal mushroom genus *Laccaria*

Authors: Andrew W. Wilson, Kentaro Hosaka, Gregory M. Mueller

Article acceptance date: 15 September 2016

The following Supporting Information is available for this article (click on underlined item to jump to page):

Notes S1a – Figure S1a: *Laccaria* Systematics phylogeny and exemplary images of *Laccaria* taxa.

S1b – Figure S1b: Global *Laccaria* phylogeny with sample IDs.

Notes S2a – Table S2a: Specimen Information and GenBank IDs

S2b – Table S2a: Agaricomycetideae dataset sequence data

S2c – Table S2c: Dataset length and composition information

Notes S3 – tMRCA priors and Agaricomycetideae phylogeny results

Notes S4a – Table S4a: Agaricomycetideae tMRCA results and comparison of molecular dating results

S4b – Table S4b: Global *Laccaria* tMRCA results

Notes S5 –BAMM results: Global *Laccaria* diversification

Notes S6 – BiSSE results: Northern vs Southern Hemisphere *Laccaria*

Notes S7 – Isotope data and specimen information.

Notes S1a

Laccaria Systematics Phylogeny (DS1)

Maximum Likelihood Tree (RAxML)

4 genes: ITS, 28S, RPB2, EF1a

(Available sequences are indicated on branch label, respectively.
Refer to Notes S2 for GenBank accession numbers.
A "0" indicates sequence not available for that gene.)

ntaxa=237 (232 ingroup)

Numbers on branches:
whole numbers = ML bootstrap % (1000 replicates)
fractions = Posterior Probabilities (10 million gen)

Branch labels in black identify samples used in tMRCA analysis of the Global *Laccaria* Dataset (Figure 1 & Notes S1b).

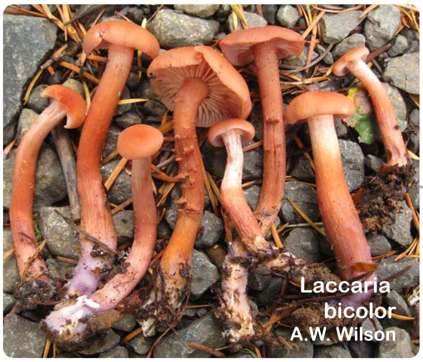

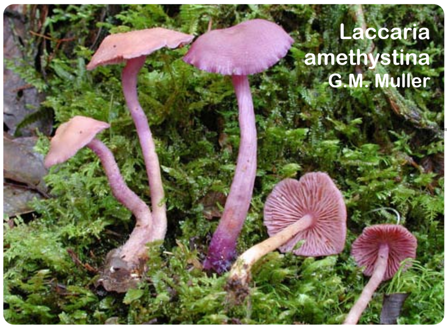

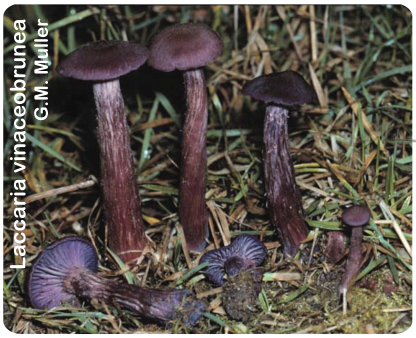

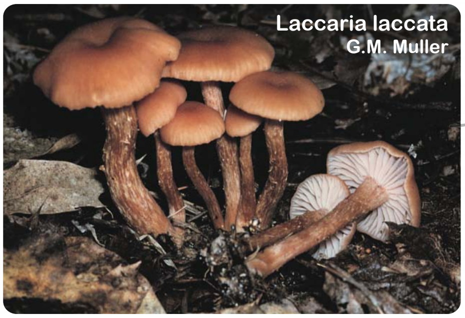

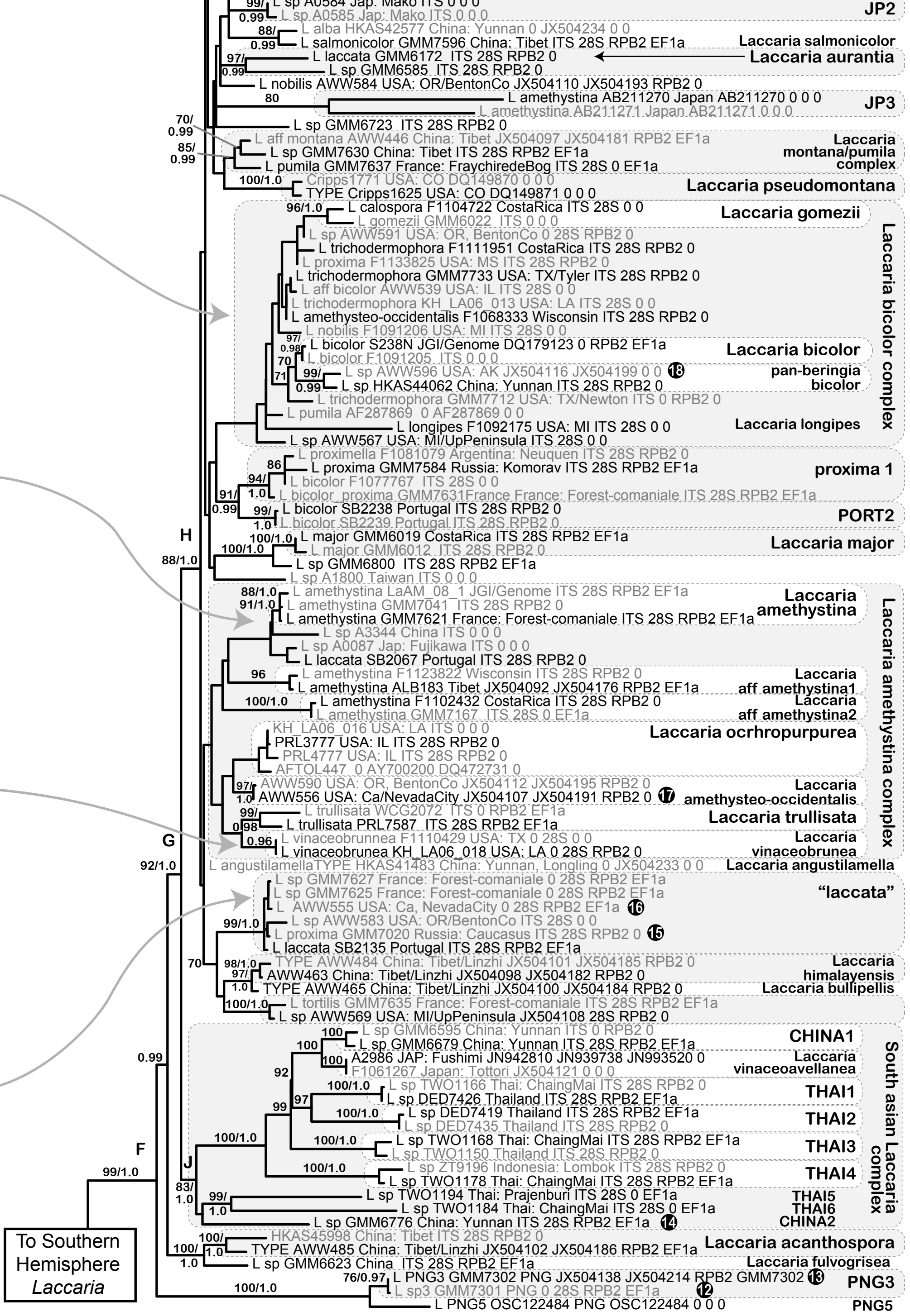

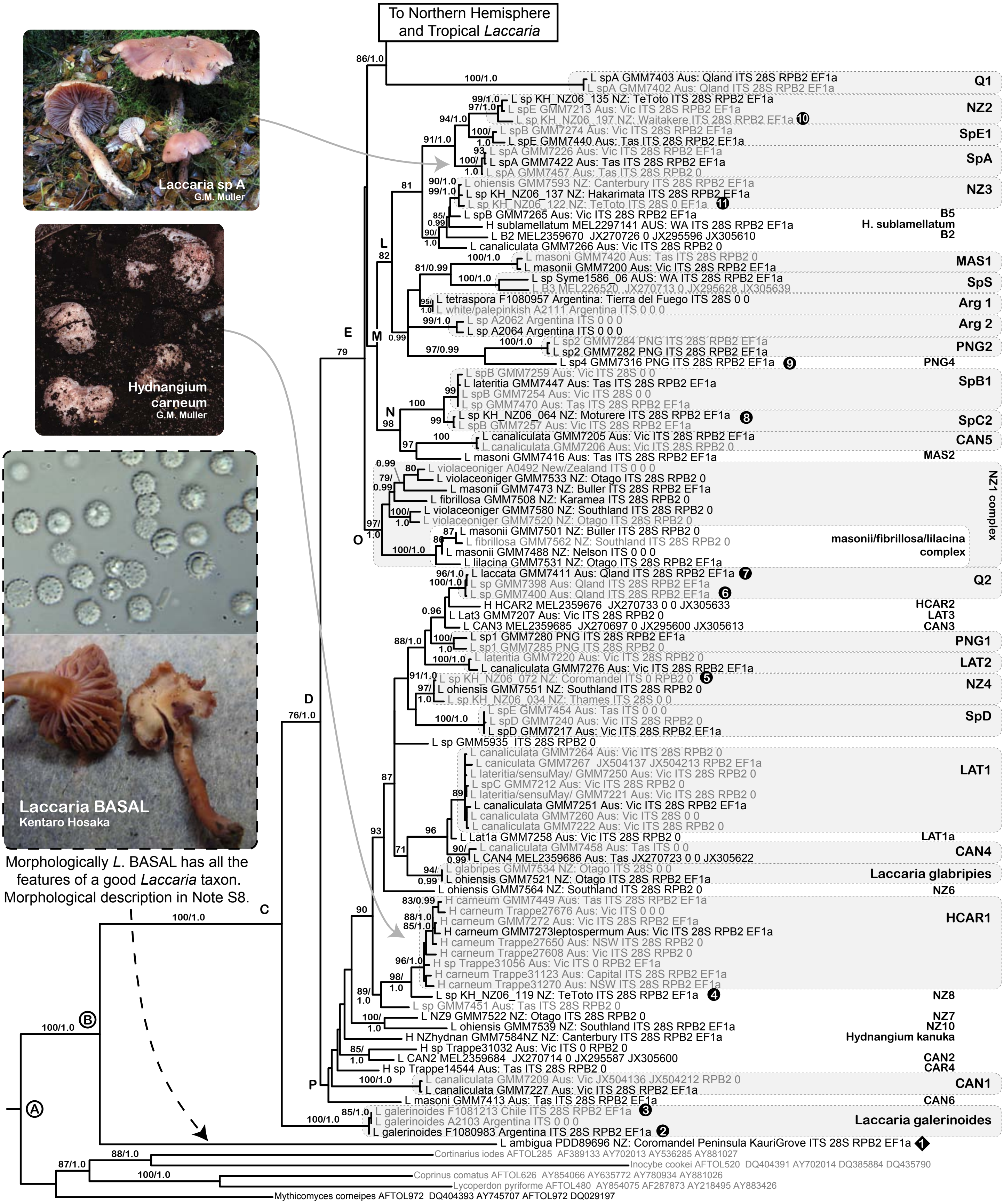

To Northern Hemisphere and Tropical Laccaria
Laccaria sp A
G.M. Muller
Hydnangium carneum
G.M. Muller
Laccaria BASAL
Kentaro Hosaka
Morphologically L. BASAL has all the features of a good Laccaria taxon. Morphological description in Note S8.
L spA GMM7403 Aus: Qland ITS 28S RPB2 EF1a
L spA GMM7402 Aus: Qland ITS 28S RPB2 EF1a
Q1
L sp KH_NZ06_135 NZ: TeToto ITS 28S RPB2 EF1a
L spE GMM7213 Aus: Vic ITS 28S RPB2 EF1a
L sp KH_NZ06_197 NZ: Waitakere ITS 28S RPB2 EF1a
NZ2
L spB GMM7274 Aus: Vic ITS 28S RPB2 EF1a
L spE GMM7440 Aus: Tas ITS 28S RPB2 EF1a
SpE1
L spA GMM7226 Aus: Vic ITS 28S RPB2 EF1a
L spA GMM7422 Aus: Tas ITS 28S RPB2 EF1a
L spA GMM7457 Aus: Tas ITS 28S RPB2 0
SpA
L ohiensis GMM7593 NZ: Canterbury ITS 28S RPB2 EF1a
L sp KH_NZ06_137 NZ: Hakarimata ITS 28S RPB2 EF1a
L sp KH_NZ06_122 NZ: TeToto ITS 28S 0 EF1a
NZ3
L spB GMM7265 Aus: Vic ITS 28S RPB2 EF1a
B5
H sublamellatum MEL2297141 AUS: WA ITS 28S RPB2 EF1a
H. sublamellatum
L B2 MEL2359670 JX270726 0 JX295596 JX305610
B2
L canaliculata GMM7266 Aus: Vic ITS 28S RPB2 0
L masoni GMM7420 Aus: Tas ITS 28S RPB2 0
L masonii GMM7200 Aus: Vic ITS 28S RPB2 EF1a
MAS1
L sp Syme1586_06 AUS: WA ITS 28S RPB2 EF1a
L B3 MEL226520 JX270713 0 JX295628 JX305639
SpS
L tetraspora F1080957 Argentina: Tierra del Fuego ITS 28S 0 0
L white/palepinkish A2111 Argentina ITS 0 0 0
Arg 1
L sp A2062 Argentina ITS 0 0 0
L sp A2064 Argentina ITS 0 0 0
Arg 2
L sp2 GMM7284 PNG ITS 28S RPB2 EF1a
L sp2 GMM7282 PNG ITS 28S RPB2 EF1a
PNG2
L sp4 GMM7316 PNG ITS 28S RPB2 EF1a
PNG4
L spB GMM7259 Aus: Vic ITS 28S 0 0
L lateritia GMM7447 Aus: Tas ITS 28S RPB2 EF1a
L spB GMM7254 Aus: Vic ITS 28S 0 0
L sp GMM7470 Aus: Tas ITS 28S RPB2 EF1a
SpB1
L sp KH_NZ06_064 NZ: Moturere ITS 28S RPB2 EF1a
L spB GMM7257 Aus: Vic ITS 28S RPB2 EF1a
SpC2
L canaliculata GMM7205 Aus: Vic ITS 28S RPB2 EF1a
L canaliculata GMM7206 Aus: Vic ITS 28S RPB2 0
CAN5
L masoni GMM7416 Aus: Tas ITS 28S RPB2 EF1a
MAS2
L violaceoniger A0492 New/Zealand ITS 0 0 0
L violaceoniger GMM7533 NZ: Otago ITS 28S RPB2 0
L masonii GMM7473 NZ: Buller ITS 28S RPB2 EF1a
L fibrillosa GMM7508 NZ: Karamea ITS 28S RPB2 0
L violaceoniger GMM7580 NZ: Southland ITS 28S RPB2 0
L violaceoniger GMM7520 NZ: Otago ITS 28S RPB2 0
L masonii GMM7501 NZ: Buller ITS 28S RPB2 0
L fibrillosa GMM7562 NZ: Southland ITS 28S RPB2 0
L masonii GMM7488 NZ: Nelson ITS 0 0 0
L lilacina GMM7531 NZ: Otago ITS 28S RPB2 EF1a
masonii/fibrillosa/lilacina complex
NZ1 complex
L laccata GMM7411 Aus: Qland ITS 28S RPB2 EF1a
L sp GMM7398 Aus: Qland ITS 28S RPB2 EF1a
L sp GMM7400 Aus: Qland ITS 28S RPB2 EF1a
Q2
H HCAR2 MEL2359676 JX270733 0 0 JX305633
HCAR2
L Lat3 GMM7207 Aus: Vic ITS 28S RPB2 0
LAT3
L CAN3 MEL2359685 JX270697 0 JX295600 JX305613
CAN3
L sp1 GMM7280 PNG ITS 28S RPB2 EF1a
L sp1 GMM7285 PNG ITS 28S RPB2 0
PNG1
L lateritia GMM7220 Aus: Vic ITS 28S RPB2 0
L canaliculata GMM7276 Aus: Vic ITS 28S RPB2 EF1a
LAT2
L sp KH_NZ06_072 NZ: Coromandel ITS 0 RPB2 0
L ohiensis GMM7551 NZ: Southland ITS 28S RPB2 0
L sp KH_NZ06_034 NZ: Thames ITS 28S 0 0
NZ4
L spE GMM7454 Aus: Tas ITS 0 0 0
L spD GMM7240 Aus: Vic ITS 28S RPB2 0
L spD GMM7217 Aus: Vic ITS 28S RPB2 EF1a
SpD
L sp GMM5935 ITS 28S RPB2 0
L canaliculata GMM7264 Aus: Vic ITS 28S RPB2 0
L caniculata GMM7267 JX504137 JX504213 RPB2 EF1a
L lateritia/sensuMay/ GMM7250 Aus: Vic ITS 28S RPB2 0
L spC GMM7212 Aus: Vic ITS 28S RPB2 0
L lateritia/sensuMay/ GMM7221 Aus: Vic ITS 28S RPB2 0
L canaliculata GMM7251 Aus: Vic ITS 28S RPB2 EF1a
L canaliculata GMM7260 Aus: Vic ITS 28S 0 0
L canaliculata GMM7222 Aus: Vic ITS 28S RPB2 0
LAT1
L Lat1a GMM7258 Aus: Vic ITS 28S RPB2 0
LAT1a
L canaliculata GMM7458 Aus: Tas ITS 0 0
L CAN4 MEL2359686 Aus: Tas JX270723 0 0 JX305622
CAN4
L glabripes GMM7534 NZ: Otago ITS 28S 0 0
L ohiensis GMM7521 NZ: Otago ITS 28S RPB2 EF1a
Laccaria glabripies
L ohiensis GMM7564 NZ: Southland ITS 28S RPB2 0
NZ6
H carneum GMM7449 Aus: Tas ITS 28S RPB2 EF1a
H carneum Trappe27676 Aus: Vic ITS 0 0 0
H carneum GMM7272 Aus: Vic ITS 28S RPB2 EF1a
H carneum GMM7273leptospermum Aus: Vic ITS 28S RPB2 EF1a
H carneum Trappe27650 Aus: NSW ITS 28S RPB2 0
H carneum Trappe27608 Aus: Vic ITS 28S RPB2 0
H sp Trappe31056 Aus: Vic ITS 0 RPB2 EF1a
H carneum Trappe31123 Aus: Capital ITS 28S RPB2 EF1a
H carneum Trappe31270 Aus: NSW ITS 28S RPB2 EF1a
HCAR1
L sp KH_NZ06_119 NZ: TeToto ITS 28S RPB2 EF1a
NZ8
L sp GMM7451 Aus: Tas ITS 28S RPB2 0
L NZ9 GMM7522 NZ: Otago ITS 28S RPB2 0
NZ7
L ohiensis GMM7539 NZ: Southland ITS 28S RPB2 EF1a
NZ10
H NZhydnan GMM7584NZ NZ: Canterbury ITS 28S RPB2 EF1a
Hydnangium kanuka
H sp Trappe31032 Aus: Vic ITS 0 RPB2 0
L CAN2 MEL2359684 JX270714 0 JX295587 JX305600
CAN2
H sp Trappe14544 Aus: Tas ITS 28S RPB2 0
CAR4
L canaliculata GMM7209 Aus: Vic JX504136 JX504212 RPB2 0
L canaliculata GMM7227 Aus: Vic ITS 28S RPB2 EF1a
CAN1
L masoni GMM7413 Aus: Tas ITS 28S RPB2 EF1a
CAN6
L galerinoides F1081213 Chile ITS 28S RPB2 EF1a
L galerinoides A2103 Argentina ITS 0 0 0
L galerinoides F1080983 Argentina ITS 28S RPB2 EF1a
Laccaria galerinoides
L ambigua PDD89696 NZ: Coromandel Peninsula KauriGrove ITS 28S RPB2 EF1a
Cortinarius iodes AFTOL285 AF389133 AY702013 AY536285 AY881027
Inocybe cookei AFTOL520 DQ404391 AY702014 DQ385884 DQ435790
Coprinus comatus AFTOL626 AY854066 AY635772 AY780934 AY881026
Lycoperdon pyriforme AFTOL480 AY854075 AF287873 AY218495 AY883426
Mythicomyces corneipes AFTOL972 DQ404393 AY745707 AFTOL972 DQ029197
A
B
C
D
E
L
M
N
O
P

Notes S1b

Global *Laccaria* Phylogeny

Bayesian Relaxed Clock Ultrametric Tree

4 genes: ITS, 28S, RPB2, EF1a

(Sample IDs are indicated on the branch labels.

Refer to Notes S2 for GenBank accession numbers.)

ntaxa=117 (116 ingroup)

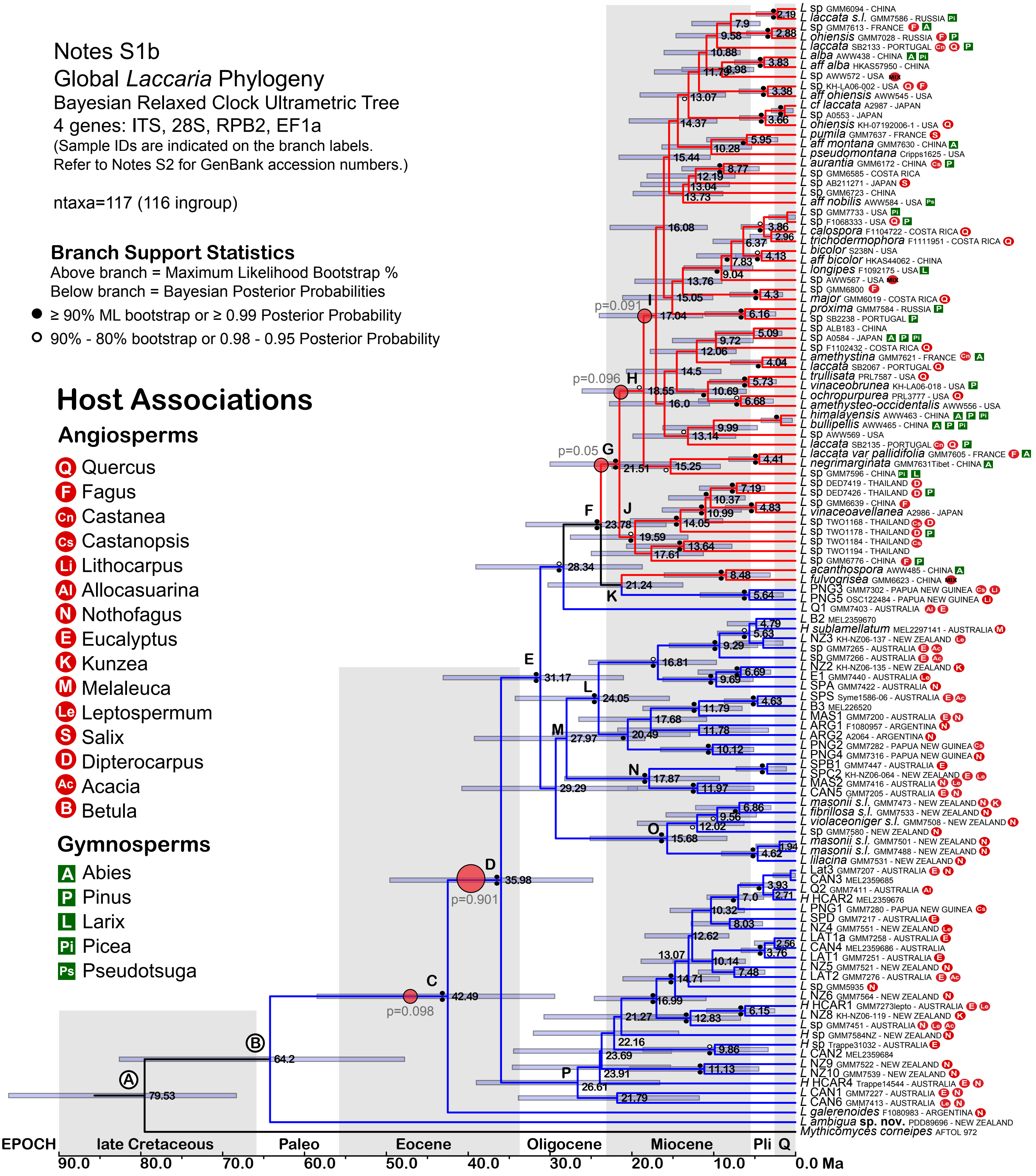

Table S2a *Laccaria* specimen IDs, geography, species assignment, and GenBank accession numbers.

**Genus species (when feasable)**	**Specimen-voucher**	**country**	**Assigned species/complex**	**ITS**	**28S**	**RPB2**	**EF1a**
Laccaria sp.	A0051	Japan: Narusawa	JP4	**KU685613**	–	–	–
Laccaria sp.	A0087	Japan: Fujikawaguchiko	Laccaria amythestina complex	**KU685614**	–	–	–
Laccaria violaceoniger	A0492	New Zealand	NZ1	**KU685615**	–	–	–
Laccaria cf. laccata	A0553	Japan: Makomanai	JP1	JN942780	JN939733	JN993511	–
Laccaria sp.	A0556	Japan: Makomanai	JP1	**KU685616**	–	–	–
Laccaria sp.	A0561	Japan: Sapporo	Laccaria alba complex	JX504082	–	–	–
Laccaria sp.	A0573	Japan: Narusawa	Laccaria alba complex	**KU685617**	–	–	–
Laccaria sp.	A0576	Japan: Tsuchiyu	JP1	**KU685618**	–	–	–
Laccaria sp.	A0577	Japan: Iwamizawa	JP1	**KU685619**	–	–	–
Laccaria sp.	A0584	Japan: Makomanai	JP2	**KU685620**	–	–	–
Laccaria sp.	A0585	Japan: Makomanai	JP2	**KU685621**	–	–	–
Laccaria sp.	A1800	Taiwan		**KU685622**	–	–	–
Laccaria sp.	A2062	Argentina	Arg2	**KU685623**	–	–	–
Laccaria sp.	A2064	Argentina	Arg2	**KU685624**	–	–	–
Laccaria galerinoides	A2103	Argentina		**KU685625**	–	–	–
Laccaria sp.	A2111	Argentina	Arg1	**KU685626**	–	–	–
Laccaria vinaceoavellanea	A2986	Japan: Fushimi		JN942810	JN939738	JN993520	–
Laccaria sp.	A2987	Japan: Fushimi	JP1	JN942786	JN939739	JN993521	–
Laccaria sp.	A3344	China	Laccaria amythestina complex	**KU685627**	–	–	–
Laccaria cf laccata	A3394	Japan: Suzuka	JP1	JN942788	JN939770	JN993522	–
Laccaria ochropurpurea	AFTOL447			–	AY700200	DQ472731	–
Laccaria alba	AWW438	China: Yunnan Shangrila	Laccaria alba complex	JX504094	JX504178	**KU685912**	**KU686072**
Laccaria himalayensis	AWW463	China: Tibet, Linzhi		JX504098	JX504182	**KU685913**	–
Laccaria bullipellis	AWW465	China: Tibet, Linzhi		JX504100	JX504184	**KU685914**	–
Laccaria himalayensis	AWW484	China: Tibet, Linzhi		JX504101	JX504185	**KU685915**	–
Laccaria acanthospora	AWW485	China: Tibet, Linzhi		JX504102	JX504186	**KU685916**	**KU686073**
Laccaria aff bicolor	AWW539	USA: Illinois	Laccaria bicolor complex	**KM067817**	**KU685763**	–	–
Laccaria tortillis	AWW545	USA: Illinois		JX504106	JX504190	**KU685917**	–
Laccaria cf laccatta	AWW555	USA: California, Nevada Co.	Laccaria laccata complex	–	**KU685764**	**KU685918**	**KU686074**
Laccaria amethysteo-occidentalis	AWW556	USA: California, Nevada Co.		JX504107	JX504191	**KU685919**	–
Laccaria sp.	AWW567	USA: Michigan, U.P.	Laccaria bicolor complex	**KM067824**	**KU685765**	–	–

Laccaria sp.	AWW569	USA: Michigan, U.P.		JX504108	**KU685766**	**KU685920**	–
Laccaria sp.	AWW572	USA: Michigan, U.P.	JP4	KM067827	**KU685767**	**KU685921**	–
Laccaria sp.	AWW583	USA: Oregon, Benton Co.	Laccaria laccata complex	KM067828	**KU685768**	–	–
Laccaria nobilis	AWW584	USA: Oregon, Benton Co.		JX504110	JX504193	**KU685922**	–
Laccaria amethysteo-occidentalis	AWW590	USA: Oregon, Benton Co.		JX504112	JX504195	**KU685923**	–
Laccaria sp.	AWW591	USA: Oregon, Benton Co.	Laccaria bicolor complex	–	**KU685769**	**KU685924**	–
Laccaria sp.	AWW596	USA: AK	pan-beringia bicolor	JX504116	JX504199	–	–
Laccaria negrimarginata	BAP360	China: Tibet		JX504120	–	–	–
Laccaria sp.	DED7419	Thailand	THAI2	**KX513839** **KX513840**	**KU685770**	**KU685925**	**KU686075**
Laccaria sp.	DED7426	Thailand	THAI1	**KU685628**	**KU685771**	**KU685926**	**KU686076**
Laccaria sp.	DED7435	Thailand	THAI2	**KX513841**	**KU685772**	**KU686060**	–
Hydnangium vinaceoavellanea	F1061267	Japan//Tottori		JX504121	–	–	–
Laccaria sp.	F1068333	USA: Wisconsin	L. bicolor complex	**KU685629**	**KU685773**	**KU686061**	–
Laccaria bicolor	GMM2692	Chile, Maullin	F1077767; proxima 1	**KU685630**	**KU685774**	–	–
Laccaria tetraspora	F1080957	Argentina: Tierra/del/Fuego	Arg1	**KU685631**	**KU685775**	–	–
Laccaria galerinoides	F1080983	Argentina		**KU685632**	**KU685776**	**KU685927**	**KU686077**
Laccaria proximella	F1081079	Argentina: Neuquen	proxima 1	**KU685633**	**KU685777**	**KU685928**	–
Laccaria galerinoides	F1081213	Chile		**KU685634**	**KU685778**	**KU685929**	**KU686078**
Laccaria bicolor	GMM2118	USA: California, Mendocino	F1091205	**KU685635**	–	–	–
Laccaria nobilis	F1091206	USA: Michigan	L. bicolor complex; "nobilis"	**KU685636**	**KU685779**	–	–
Laccaria longipes	F1092175	USA: Michigan		**KU685637**	**KU685780**	–	–
Laccaria sp.	F1102432	Costa Rica	L. aff amythestina 2	**KU685638**	**KU685781**	**KU686062**	–
Laccaria gomezii	F1104722	Costa Rica		**KU685639**	**KU685782**	–	–
Laccaria vinaceobrunnea	F1110429	USA: Texas		–	**KU685783**	–	–
Laccaria sp.	F1111951	Costa Rica	L. "trichodermophora"	**KU685640**	**KU685784**	**KU686063**	–
Laccaria tortilis	F1116205	USA: Illinois		**KU685641**	**KU685785**	–	–
Laccaria sp.	F1120731	China	JP1	JX504125	JX504205	–	–
Laccaria alba	F1120750	China	L. alba complex	JX504126	JX504242	–	–
Laccaria bicolor	F1121424	China		JX504127	JX504207	**KU686064**	–
Laccaria proxima	F1133825	USA: Mississippi	L. bicolor complex	**KU685642**	**KU685786**	**KU686065**	–
Laccaria sp.	GMM5935	New Zealand		**KU685643**	**KU685787**	**KU686066**	–
Laccaria gomezii	GMM6022	Costa Rica		**KU685644**	–	–	–

Laccaria bicolor	GMM6094	China: Jilin Province		KM067831	**KU685788**	**KU686067**	–
Laccaria alba	GMM6131	China: Chang Bai Shan		JX504131	JX504210	**KU685930**	**KU686079**
Laccaria aurantia	GMM6172	China: Yunnan		**KU685645**	**KU685789**	**KU685931**	–
Laccaria fulvogrisea	GMM6623	China		**KU685646**	**KU685790**	**KU685932**	**KU686080**
Laccaria sp.	GMM6585	Costa Rica		**KU685647**	**KU685791**	**KU685933**	–
Laccaria sp.	GMM6595	China: Yunnan	CHINA1	**KU685648**	–	**KU685934**	–
Laccaria sp.	GMM6679	China: Yunnan	CHINA1	**KU685649**	**KU685792**	**KU685935**	**KU686081**
Laccaria sp.	GMM6723	China: Yunnan	N25° 40.566' E 98° 42.166'	**KU685650**	**KU685793**	**KU685936**	–
Laccaria sp.	GMM6776	China: Yunnan	CHINA2	**KU685651**	**KU685794**	**KU685937**	**KU686082**
Laccaria sp.	GMM7020	Russia: Caucasus	"laccata"	**KU685652**	**KU685795**	**KU685938**	–
Laccaria sp.	GMM7028	Russia: Caucasus	L. macrocystidia complex	**KU685653**	**KU685796**	**KU685939**	–
Laccaria amethystina	GMM7041	Russia: Caucasus		**KU685654**	**KU685797**	**KU685940**	–
Laccaria sp.	GMM7167	Costa Rica	L. aff amythestina 2	**KU685655**	**KU685798**	–	**KU686083**
Laccaria masonii	GMM7200	Australia: Victoria	MAS1	**KU685656**	**KU685799**	**KU685941**	**KU686084**
Laccaria canaliculata	GMM7205	Australia: Victoria	CAN5	**KU685657**	**KU685800**	**KU685942**	**KU686085**
Laccaria canaliculata	GMM7206	Australia: Victoria	CAN5	**KU685658**	**KU685801**	**KU685943**	–
Laccaria canaliculata	GMM7209	Australia: Victoria	CAN1	JX504136	JX504212	**KU685944**	–
Laccaria sp.	GMM7212	Australia: Victoria	sp.C	**KU685659**	**KU685802**	**KU685945**	–
Laccaria sp.	GMM7213	Australia: Victoria	sp.E	**KU685660**	**KU685803**	**KU685946**	**KU686086**
Laccaria sp.	GMM7217	Australia: Victoria	sp.D	**KU685661**	**KU685804**	**KU685947**	**KU686087**
Laccaria lateritia	GMM7220	Australia: Victoria	LAT2	**KU685662**	**KU685805**	**KU685948**	–
Laccaria lateritia	GMM7221	Australia: Victoria	LAT1	**KU685663**	**KU685806**	**KU685949**	–
Laccaria canaliculata	GMM7222	Australia: Victoria	LAT1	**KU685664**	**KU685807**	**KU685950**	–
Laccaria sp.	GMM7226	Australia: Victoria	sp.A	**KU685665**	**KU685808**	**KU685951**	**KU686088**
Laccaria canaliculata	GMM7227	Australia: Victoria	CAN1	**KU685666**	**KU685809**	**KU685952**	**KU686089**
Laccaria sp.	GMM7240	Australia: Victoria	sp.D	**KU685667**	**KU685810**	**KU685953**	–
Laccaria lateritia	GMM7250	Australia: Victoria	LAT2	**KU685668**	**KU685811**	**KU685954**	–
Laccaria canaliculata	GMM7251	Australia: Victoria	LAT1	**KU685669**	**KU685812**	**KU685955**	**KU686090**
Laccaria sp.	GMM7254	Australia: Victoria	sp.B1	**KU685670**	**KU685813**	–	–
Laccaria sp.	GMM7257	Australia: Victoria	sp.C2	**KU685671**	**KU685814**	**KU685956**	**KU686091**
Laccaria sp.	GMM7259	Australia: Victoria	sp.B1	**KU685672**	**KU685815**	–	–
Laccaria canaliculata	GMM7260	Australia: Victoria	LAT1	**KU685673**	**KU685816**	–	–
Laccaria canaliculata	GMM7264	Australia: Victoria	LAT1	**KU685674**	**KU685817**	**KU685957**	–
Laccaria sp.	GMM7265	Australia: Victoria	sp.B5	**KU685675**	**KU685818**	**KU685958**	**KU686092**

Laccaria sp.	GMM7266	Australia: Victoria	"canaliculata"	**KU685676**	**KU685819**	**KU685959**	–
Laccaria caniculata	GMM7267	Australia: Victoria	LAT1	JX504137	JX504213	**KU685960**	**KU686093**
Hydnangium carneum	GMM7272	Australia: Victoria	HCAR1	**KU685677**	**KU685820**	**KU685961**	**KU686094**
Hydnangium carneum	GMM7273lepto.	Australia: Victoria	HCAR1	**KU685678**	**KU685821**	**KU685962**	**KU686095**
Laccaria sp.	GMM7274	Australia: Victoria	sp.E1	**KU685679**	**KU685822**	**KU685963**	**KU686096**
Laccaria canaliculata	GMM7276	Australia: Victoria	LAT2	**KU685680**	**KU685823**	**KU685964**	**KU686097**
Laccaria PNG1	GMM7280	Papua New Guinea	PNG1	**KU685681**	**KU685824**	**KU685965**	**KU686098**
Laccaria PNG2	GMM7282	Papua New Guinea	PNG2	**KU685682**	**KU685825**	**KU685966**	**KU686099**
Laccaria PNG2	GMM7284	Papua New Guinea	PNG2	**KU685683**	**KU685826**	**KU685967**	**KU686100**
Laccaria PNG1	GMM7285	Papua New Guinea	PNG1	**KU685684**	**KU685827**	**KU685968**	–
Laccaria PNG3	GMM7301	Papua New Guinea	PNG3	–	**KU685828**	**KU685969**	**KU686101**
Laccaria sp.	GMM7302	Papua New Guinea	PNG3	JX504138	JX504214	**KU685970**	**KU686102**
Laccaria PNG4	GMM7316	Papua New Guinea	PNG4	**KU685685**	**KU685829**	**KU685971**	**KU686103**
Laccaria sp.	GMM7398	Australia: Queensland	Q2	**KU685686**	**KU685830**	**KU685972**	**KU686104**
Laccaria sp.	GMM7400	Australia: Queensland	Q2	**KU685687**	**KU685831**	**KU685973**	**KU686105**
Laccaria sp.	GMM7402	Australia: Queensland	Q1	**KU685688**	**KU685832**	**KU685974**	**KU686106**
Laccaria sp.	GMM7403	Australia: Queensland	Q1	**KU685689**	**KU685833**	**KU685975**	**KU686107**
Laccaria laccata	GMM7411	Australia: Queensland	Q2	**KU685690**	**KU685834**	**KU685976**	**KU686108**
Laccaria masoni	GMM7413	Australia: Tasmania	CAN1	**KU685691**	**KU685835**	**KU685977**	**KU686109**
Laccaria masoni	GMM7416	Australia: Tasmania	MAS2	**KU685692**	**KU685836**	**KU685978**	**KU686110**
Laccaria masoni	GMM7420	Australia: Tasmania	MAS1	**KU685693**	**KU685837**	**KU685979**	–
Laccaria sp.A	GMM7422	Australia: Tasmania	sp.A	**KU685694**	**KU685838**	**KU685980**	**KU686111**
Laccaria sp.E	GMM7440	Australia: Tasmania	sp.E	**KU685695**	**KU685839**	**KU685981**	**KU686112**
Laccaria lateritia	GMM7447	Australia: Tasmania	sp.B1	**KU685696**	**KU685840**	**KU685982**	**KU686113**
Hydnangium carneum	GMM7449	Australia: Tasmania	HCAR1	**KU685697**	**KU685841**	**KU685983**	**KU686114**
Laccaria sp.	GMM7451	Australia: Tasmania		**KU685698**	**KU685842**	**KU685984**	–
Laccaria sp.E	GMM7454	Australia: Tasmania	sp.E	**KU685699**	–	–	–
Laccaria sp.A	GMM7457	Australia: Tasmania	sp.A	**KU685700**	**KU685843**	**KU685985**	–
Laccaria canaliculata	GMM7458	Australia: Tasmania	CAN4	**KU685701**	–	–	–
Laccaria sp.	GMM7470	Australia: Tasmania	sp.B1	**KU685702**	**KU685844**	**KU685986**	**KU686115**
Laccaria masonii	GMM7473	New Zealand: Buller	NZ1complex	**KU685703**	**KU685845**	**KU685987**	**KU686116**
Laccaria masonii	GMM7488	New Zealand: Nelson	masonii/fibrillosa/lilacina complex	**KU685704**	–	–	–
Laccaria masonii	GMM7501	New Zealand: Buller	masonii/fibrillosa/lilacina complex	**KU685705**	**KU685846**	**KU685988**	–

Laccaria fibrillosa	GMM7508	New Zealand: Karamea	NZ1complex	**KU685706**	**KU685847**	**KU685989**	–
Laccaria violaceoniger	GMM7520	New Zealand: Otago	NZ1complex	**KU685707**	**KU685848**	**KU685990**	–
Laccaria glabripies	GMM7521	New Zealand: Otago		**KU685708**	**KU685849**	**KU685991**	**KU686117**
Laccaria lilacina	GMM7531	New Zealand: Otago	masonii/fibrillosa/lilacina complex	**KU685709**	**KU685850**	**KU685992**	**KU686118**
Laccaria violaceoniger	GMM7533	New Zealand: Otago	NZ1complex	**KU685710**	**KU685851**	**KU685993**	–
Laccaria glabripes	GMM7534	New Zealand: Otago		**KU685711**	**KU685852**	–	–
Laccaria ohiensis	GMM7539	New Zealand: Southland	NZ10	**KU685712**	**KU685853**	**KU685994**	**KU686119**
Laccaria ohiensis	GMM7551	New Zealand: Southland	NZ4	**KU685713**	**KU685854**	**KU685995**	–
Laccaria fibrillosa	GMM7562	New Zealand: Southland	masonii/fibrillosa/lilacina complex	**KU685714**	**KU685855**	**KU685996**	–
Laccaria ohiensis	GMM7564	New Zealand: Southland	NZ6	**KU685715**	**KU685856**	**KU685997**	–
Laccaria violaceoniger	GMM7580	New Zealand: Southland	NZ1complex	**KU685716**	**KU685857**	**KU685998**	–
Laccaria proxima	GMM7584	Russia: Komorav	proxima 1	**KU685717**	**KU685858**	**KU685999**	**KU686120**
Laccaria laccata	GMM7586	Russia: Komorav	Russia-China group?	KM067835	**KU685859**	**KU686000**	–
Laccaria ohiensis	GMM7493NZ	New Zealand: Canterbury	NZ3	**KU685718**	**KU685860**	**KU686001**	**KU686121**
Laccaria macrocystidia	GMM7612	France: Vallon		KM067847	**KU685861**	**KU686002**	**KU686122**
Laccaria macrocystidia	GMM7613	France: Vallon		KM067848	**KU685862**	**KU686003**	**KU686123**
Laccaria macrocystidia	GMM7616	France: Vallon		KM067850	**KU685863**	**KU686004**	–
Laccaria sp.	GMM7625	France: Forest comaniale	"laccata"	–	**KU685864**	**KU686005**	**KU686124**
Laccaria macrocystidia	GMM7626	France: Forest comaniale		KM067856	**KU685865**	**KU686006**	**KU686125**
Laccaria sp.	GMM7627	France: Forest comaniale	"laccata"	–	**KU685866**	**KU686007**	**KU686126**
Laccaria macrocystidia	GMM7628	France: Forest comaniale		KM067857	**KU685867**	**KU686008**	**KU686127**
Laccaria sp.	GMM7630tibet	China: Tibet	montana/pumula complex	JX504151	JX504225	**KU686009**	**KU686128**
Laccaria bicolor/proxima	GMM7631France	France: Forest comaniale	proxima 1	KM067858	**KU685869**	**KU686010**	**KU686129**
Laccaria negrimarginata	GMM7631Tibet	China: Tibet		JX504152	JX504226	**KU686011**	**KU686130**
Laccaria trichodermophora	GMM7712	USA: Texas, Newton Co.	L. bicolor complex	KM067866	–	**KU686012**	–
Laccaria trichodermophora	GMM7733	USA: Texas, Tyler Co.	L. "trichodermophora"	JX504157	JX504230	**KU686013**	–
Laccaria alba	HKAS41457	China: Yunnan Longling		–	JX504232	–	–
Laccaria angustilamella	HKAS41483	China: Yunnan Longling		–	JX504233	–	–
Laccaria sp.	HKAS42577	China: Yunnan	specimen named "alba"	–	JX504234	–	–
Laccaria sp.	HKAS44062	China: Yunnan	pan-beringia bicolor	JX504159	JX504235	**KU686068**	–
Laccaria acanthospora	HKAS45998	China: Tibet		**KU685719**	**KU685870**	**KU686069**	–
Laccaria sp.	HKAS57950	China: Tibet	L. alba complex	JX504166	JX504242	–	–
Laccaria ohiensis	KH_07192006_1	USA: Indiana		**KU685720**	**KU685871**	**KU686014**	–

Laccaria trichodermophora	KH_LA06_013	USA: Louisiana	L. "trichodermophora"	KM067881	**KU685872**	–	–
Laccaria ochropurpurea	KH_LA06_016	USA: Louisiana		**KU685721**	–	–	–
Laccaria vinaceobrunea	KH_LA06_018	USA: Louisiana		–	**KU685873**	**KU686015**	–
Laccaria sp.	PDD 89649	New Zealand: Thames	NZ4	**KU685722**	**KU685874**	–	–
Laccaria sp.	PDD 89677	New Zealand: Moturere	sp.C2	**KU685723**	**KU685875**	**KU686016**	**KU686131**
Laccaria sp.	PDD 89685	New Zealand: Coromandel	NZ4	**KU685724**	–	**KU686017**	–
Laccaria ambigua	PDD 89696	New Zealand: Kauri Grove	L ambigua	**KU685725**	**KU685876**	**KU686018**	**KU686132**
Laccaria sp.	PDD 89737	New Zealand: TeToto	NZ8	**KU685726**	**KU685877**	**KU686019**	**KU686133**
Laccaria sp.	PDD 89739	New Zealand: TeToto	NZ3	**KU685727**	**KU685878**	–	**KU686134**
Laccaria sp.	PDD 89750	New Zealand: TeToto	NZ2	**KU685728**	**KU685879**	**KU686020**	**KU686135**
Laccaria sp.	PDD 89752	New Zealand: Hakarimata	NZ3	**KU685729**	**KU685880**	**KU686021**	**KU686136**
Laccaria sp.	PDD 89812	New Zealand: Waitakere	NZ2	**KU685730**	**KU685881**	**KU686022**	**KU686137**
Laccaria amethystina	LaAM-08-1	JGI/Genome		JGI	JGI	JGI	JGI
Hydnangium sublamellatum	MEL2297141	Australia: Western Australia		**KU685731**	**KU685882**	**KU686023**	**KU686138**
Laccaria sp.	MEL2359686	Australia: Tasmania	CAN4	JX270723	–	–	JX305622
Laccaria sp.	MEL2359670		B2	JX270726	–	JX295596	JX305610
Laccaria sp.	MEL226520		B3	JX270713	–	JX295628	JX305639
Laccaria sp.	MEL2359685		CAN3	JX270697	–	JX295600	JX305613
Hydnangium HCAR2	MEL2359676		HCAR2	JX270733	–	–	JX305633
Laccaria sp.	MEL2359684		CAN2	JX270714	–	JX295587	JX305600
Laccaria ochropurpurea	PRL3777	USA: Illinois		**KU685732**	JX504246	**KU686024**	–
Laccaria ochropurpurea	PRL4777	USA: Illinois		**KU685733**	**KU685883**	**KU686025**	–
Laccaria bicolor	S238N	JGI/Genome		DQ179123	–	JGI	JGI
Laccaria sp.	SB2067	Portugal	L. amythestina complex	KM067885	JX504248	**KU686026**	–
Laccaria sp.	SB2133	Portugal	PORT1	KM067887	**KU685884**	**KU686027**	**KU686139**
Laccaria sp.	SB2135	Portugal	"laccata"	JX504172	JX504249	**KU686028**	**KU686140**
Laccaria sp.	SB2210	Portugal	PORT1	KM067890	**KU685885**	**KU686029**	**KU686141**
Laccaria bicolor	SB2238	Portugal	PORT2	KM067892	**KU685886**	**KU686030**	–
Laccaria bicolor	SB2239	Portugal	PORT2	KM067893	**KU685887**	**KU686031**	–
Laccaria sp.	Syme1586/06	Australia: Western Australia	sp.S	**KU685734**	**KU685888**	**KU686032**	**KU686142**
Hydnangium sp.	Trappe14544	Australia: Tasmania	CAR4	**KU685735**	**KU685889**	**KU686033**	–
Hydnangium carneum	Trappe27608	Australia: Victoria	HCAR1	**KU685736**	**KU685890**	**KU686034**	–
Hydnangium carneum	Trappe27650	Australia: New South Wales	HCAR1	**KU685737**	**KU685891**	**KU686035**	–

Hydnangium carneum	Trappe27676	Australia: Victoria	HCAR1	**KU685738**	–	–	–
Hydnangium sp.	Trappe31032	Australia: Victoria		**KU685739**	–	**KU686036**	–
Hydnangium sp.	Trappe31056	Australia: Victoria	HCAR1	**KU685740**	–	**KU686037**	**KU686143**
Hydnangium carneum	Trappe31123	Australia: Capital	HCAR1	**KU685741**	**KU685892**	**KU686038**	**KU686144**
Hydnangium carneum	Trappe31270	Australia: New South Wales	HCAR1	**KU685742**	**KU685893**	**KU686039**	**KU686145**
Laccaria sp.	TWO1150	Thailand	THAI3	**KU685743**	**KU685894**	**KU686040**	–
Laccaria sp.	TWO1166	Thailand: Chaing Mai	THAI1	**KU685744**	**KU685895**	**KU686041**	–
Laccaria sp.	TWO1168	Thailand: Chaing Mai	THAI3	**KU685745**	**KU685896**	**KU686042**	**KU686146**
Laccaria sp.	TWO1178	Thailand: Chaing Mai	THAI4	**KU685746**	**KU685897**	**KU686043**	**KU686147**
Laccaria sp.	TWO1184	Thailand: Chaing Mai	THAI6	**KU685747**	**KU685898**	–	**KU686148**
Laccaria sp.	TWO1194	Thailand: Prajenburi	THAI5	**KU685748**	**KU685899**	–	**KU686149**
Laccaria trullisata	WCG2072			**KU685749**	–	**KU686044**	**KU686150**
Laccaria sp.	ZT9196	Indonesia: Lombok	THAI4	**KU685750**	**KU685900**	**KU686070**	–
Laccaria salmonicolor	GMM7596tibet	China: Tibet		JX504143	JX504218	**KU686045**	**KU686151**
Laccaria amethystina	GMM7621	France: Forest comaniale		JX504150	JX504224	**KU686046**	**KU686152**
Laccaria trullisata	PRL7587			KM067882	JX504247	**KU686047**	**KU686153**
Laccaria laccata var. pallidifolia	GMM7605	France: Vallon		KM067844	**KU685901**	**KU686048**	**KU686154**
Laccaria sp.	GMM7258	Australia: Victoria	Lat1a	**KU685751**	**KU685902**	**KU686049**	–
Laccaria sp.	GMM7207	Australia: Victoria	Lat3	**KU685752**	**KU685903**	**KU686050**	–
Laccaria sp.	GMM7522	New Zealand: Otago	NZ9	**KU685753**	**KU685904**	**KU686051**	–
Hydnangium sp.	GMM7484NZ	New Zealand: Canterbury	NZhydnan	**KU685754**	**KU685905**	**KU686052**	**KU686155**
Laccaria sp.	OSC122484	Papua New Guinea	PNG5	**KU685755**	–	–	–
Laccaria pumila	AF287869			–	AF287869	–	–
Laccaria tortilis	GMM7635	France: Forest comaniale		KM067859	**KU685906**	**KU686053**	**KU686156**
Laccaria aff. montana	AWW446	China: Tibet	montana/pumula complex	JX504097	JX504181	**KU686054**	**KU686157**
Laccaria pumila	GMM7637	France: Fraychirede Bog	montana/pumula complex	KM067861	JX504229	–	**KU686158**
Laccaria pseudomontana	Cripps1625	USA: Colorado		DQ149871	–	–	–
Laccaria pseudomontana	Cripps1771	USA: Colorado		DQ149870	–	–	–
Laccaria sp.	GMM6800	Guatemala		**KU685756**	**KU685907**	**KU686055**	**KU686159**
Laccaria major	GMM6019	Costa Rica		**KU685757**	**KU685908**	**KU686056**	**KU686160**
Laccaria major	GMM6012	Costa Rica		**KU685758**	**KU685909**	**KU686057**	–
Laccaria sp.	ALB183	China: Tibet	Laccaria aff amythestina 1	JX504092	JX504176	**KU686058**	**KU686161**
Laccaria amethystina	KH_LA06_002	USA: Louisiana		**KU685759**	**KU685910**	**KU686059**	**KU686162**
Laccaria sp.	F1123822	USA: Wisconsin	Laccaria aff amythestina 1	**KU685760**	**KU685911**	**KU686071**	–
Laccaria amethystina	AB211270	Japan		AB211270	–	–	–
Laccaria amethystina	AB211271	Japan		AB211271	–	–	–

Laccaria sp.	A4296	New Caledonia	CAL	**KU685761**	–	–	–
Laccaria sp.	A4366	New Caledonia	CAL	**KU685762**	–	–	–
Coprinus comatus	AFTOL626			AY854066	AY635772	AY780934	AY881026
Cortinarius iodes	AFTOL285			AF389133	AY702013	AY536285	AY881027
Inocybe cookei	AFTOL520			DQ404391	AY702014	DQ385884	DQ435790
Lycoperdon pyriforme	AFTOL480			AY854075	AF287873	AY218495	AY883426
Mythicomyces corneipes	AFTOL972			DQ404393	AY745707	AFTOL972	DQ029197

Table S2b Agaricomycetideae dataset.

**Genus species**	**Sample ID**	**Taxonomic group**	**18S**	**28S**	**RPB1**	**RPB2**	**EF1a**
Coniophora arida	AFTOL 698	Boletales	AY293123	-	GU187492	DQ366282	DQ408146
Suillus pictus	AFTOL 717	Boletales	AY662659	AY684154	AY858965	AY786066	AY883429
Gomphidius roseus	AFTOL 1780	Boletales	DQ534682	DQ534669	GU187459	GU187818	GU187702
Mycena amabilissima	AFTOL 1686	Agaricomycotina	DQ457647	DQ457691	DQ447926	DQ474121	GU187727
Marasmius rotula	AFTOL 1505	Agaricomycotina	DQ113912	DQ457686	DQ447922	DQ474118	GU187723
Fibularhizoctonia sp	AFTOL 576	Athelioid (root)	AY654887	AY635779	AY857985	AY885161	AY879115
Inocybe cookei	AFTOL 520	Agaricomycotina	AY752967	AY702014	DQ447915	DQ385884	DQ435790
Inocybe dulcamara	AFTOL 482	Agaricomycotina	AY657016	AY700196	DQ447916	AY803751	DQ435791
Cortinarius iodes	AFTOL 285	Cortinariaceae	AY771605	AY702013	AY857984	AY536285	AY881027
Cortinarius aurilicis	AFTOL 812	Cortinariaceae	AY705957	AY684152	DQ083826	DQ083880	DQ061278
Galerina semilanceata	PBM 1398	Hymenogastraceae	DQ440639	AY038309	AF389531	AY337357	-
Hebeloma velutipes	AFTOL 980	Hymenogastraceae	AY752972	AY745703	DQ447904	DQ472718	-
Hebeloma olympianum	BK21_Nov_8_20/UTC/	Hymenogastraceae	-	AY038310	AF389532	AY337359	-
Agrocybe praecox	AFTOL 728	Strophariaceae	AY705956	AY646101	DQ516069	DQ385876	DQ061276
Hyphaloma fasciculare	PBM 1844	Strophariaceae	-	AY380409	AY351829	AY337413	-
Tubaria confragosa	AFTOL 498	Tubareae	AY665776	AY700190	DQ447944	DQ408113	-
Bolbitius vitellinus	AFTOL 730	Bolbitiaceae	AY705955	AY691807	DQ435802	DQ385878	DQ408148
Lycoperdon pyriforme	AFTOL 480	Agaricaceae	AF026619	AF287873	AY860524	AY218495	AY883426
Coprinus comatus	AFTOL 626	Agaricaceae	AY665772	AY635772	AY857983	AY780934	AY881026
Mythicomyces corneipes	AFTOL 972	Psathrellaceae	DQ092917	AY745707	DQ447929	Unpublished	DQ029197
Coprinopsis cinerea	MIX	Psathrellaceae	GenomeM92991	AF041494	Genome	Genome	-
Lacrymaria velutina	AFTOL 478	Psathrellaceae	AY654885	AY700198	-	DQ472733	-
Laccaria amethystina	LaAM-08-1	Laccaria (ingroup)	JGI	-	JGI	JGI	-
Laccaria galerinoides	F1080983	Laccaria (ingroup)	-	KU685776	-	KU685927	KU686077
Laccaria bicolor	S238	Laccaria (ingroup)	JGI	JGI	JGI	JGI	-
Laccaria ochropurpurea	AFTOL 477	Laccaria (ingroup)	AY654886	AY700200	-	DQ472731	-
Laccaria ambigua	PDD 89696	Laccaria (ingroup)	-	KU685876	-	KU686018	KU686132
Laccaria carneum	GMM7272	Laccaria (ingroup)	-	KU685820	-	KU685961	KU686094
Laccaria acanthospora	AWW485	Laccaria (ingroup)	-	JX504186	-	KU685916	KU686073

Laccaria Thailand	DED7426	Laccaria (ingroup)	-	KU685771	-	KU685926	KU686076
Laccaria alba	AWW438	Laccaria (ingroup)	-	JX504178	-	KU685912	KU686072
Laccaria canaliculata	GMM7205	Laccaria (ingroup)	-	KU685800	-	KU685942	KU686085
Laccaria masonii	GMM7200	Laccaria (ingroup)	-	KU685799	-	KU685941	KU686084
Laccaria lilacina	GMM7531	Laccaria (ingroup)	-	KU685850	-	KU685992	KU686118
Laccaria spA	GMM7402	Laccaria (ingroup)	-	KU685832	-	KU685974	KU686106

Table S2c Dataset length statistics.

**Dataset**	**Total Taxa**	**Total Char.**	**18S**	**ITS**	**28S**	**RPB2**	**RPB1**	**EF1a**
Laccaria Systematics	237	3755	NA	780	768	1091	NA	1116
Global *Laccaria**	116	3243	NA	676	732	1010	NA	825
Agaricomycetideae*	35	6626	1685	NA	1351	2075	573	942

*=introns removed from protein sequences
NA = region not used for dataset

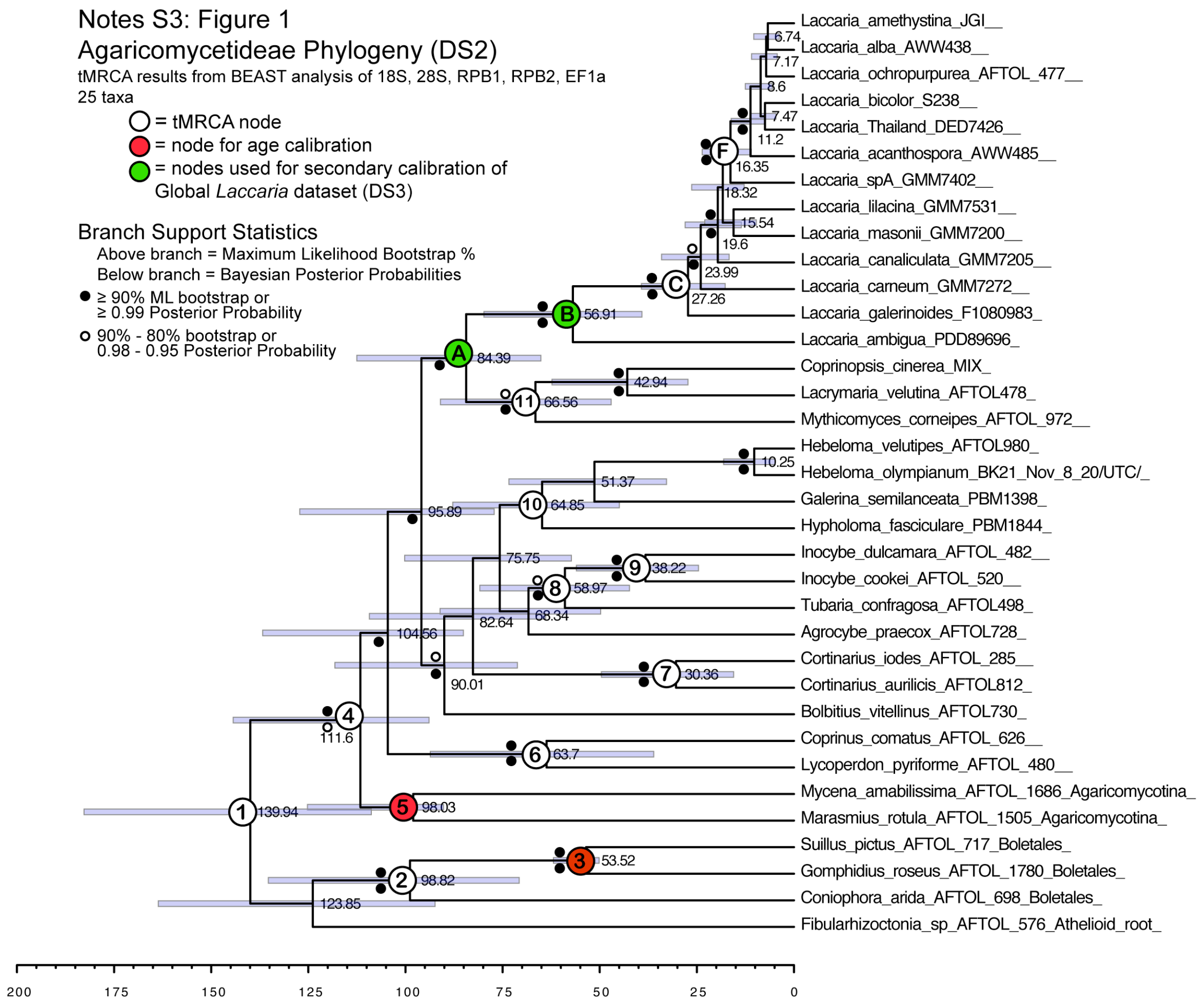

Agaricomycetideae Dataset (Notes S3 Figure 1)

tMRCA	Node	Distribution	Initial Value	Calibration Mean*	Prior Mean**	Log (Stdev)	Offset	Mean in Real Space	Reference
marasmioid	5	Lognormal		92	10	1	90	yes	Hibbett et al. 1997
Suillineae	3	Lognormal		55	10	1	50	yes	LePage et al. 1997

Global *Laccaria* Dataset (Fig. 1)

tMRCA	Node	Distribution	Initial Value	Calibration Mean*	Prior Mean**	Log (Stdev)	Offset	Mean in Real Space	Reference
root	A	Lognormal	87	86.94	26	0.5	63	yes	Floudas et al. 2012 (node 15)
Hydnangiaceae	B	Lognormal	58	50.19	23	0.5	37	yes	Agaricomycetideae Dataset

* This is the mean age, based on literature/analysis, used to calibrate the dataset

** This is the mean prior supplied to BEAUTi to approximate the Calibration Mean

Notes S4 – Results of tMRCA analyses Wilson et al. *Laccaria* evolution

Table S4a Dating results and age comparisons for Step 1 – Agaricomycetideae Dataset. (Notes S3)

Total Trees = 18002

likelihood = -44883.682 (ESS 1620)

						Comparison to Floudas et al. 2012								
**Node**	**tMRCA**	**Mean**	**Median**	**95% CI**	**ESS**	**Node**	**AGA BOL ASC**	**95% CI**	**AGA BOL**	**95% CI**	**AGA ASC**	**95% CI**	**BOL ASC**	**95% CI**
1	Root - Agaricomycetideae	145.8	143.59	117.7-179.23	1285	13	148.94	117.17-187.56	139.6	109.89-176.71	152.95	117.84-194.63	140.42	102.64-185.11
2	Boletales	101.72	98.86	71.62-136.72	479	16	104.23	73.93-138.7	97.62	70.03-129.44	110.18	78.12-147.24	100.35	67.64-136.98
3*	Suillineae	54.57	53.52	50.16-61.90	4112									
4	Agaricales	115.06	111.68	93.52-144.35	739	14	122.74	97.46-154.27	115.48	94.6-144.78	125.12	98.12-158.97	114.1	80.37-154.32
5*	Marasmiaceae	101.83	98.03	90.19-125.22	1141									
6	Agariceae	64.47	63.70	36.10-93.59	391									
7	Cortinariaceae	31.75	30.36	15.53-49.57	413									
8	Inocybaceae + Tubariaceae	60.09	58.97	42.32-80.91	606									
9	Inocybaceae	39.02	38.22	24.56-56.01	299									
10	Strophariaceae	77.06	75.53	56.05-100.21	659									
11	Psathyrellaceae	67.84	66.56	47.08-90.98	708									
A**	Laccaria+ Psathyrellaceae	86.26	84.39	65.16-112.51	731	15	86.94	62.65-115.18	82.39	59.38-108.4	86.89	61.6-117.1	81.09	51.78-114.07
B**	Laccaria (=Hydnangiaceae)	58.05	56.90	39.14-79.82	786									
C	Laccaria s.s. minus Laccaria ambigua		27.26											
F	NH Laccaria	11.48	11.20	7.34-16.17	242									

* = Node used in calibration of the dataset.
** = tMRCA results used to calibrate step 2 analysis.
ESS = effective sample size. A value >200 is the target for effective statistical sampling.

Table S4b Dating results for Step 2 - Global *Laccaria* Dataset. (Figure 1)

Total Trees = 49756
likelihood = -28609.301 (ESS 2851)

Node	tMRCA	Mean	Median	95% CI	ESS
A*	Root = Laccaria+ Mythicomyces	80.89	79.53	68.32-96.14	13367
B*	Laccaria	64.78	64.20	47.7-82.62	1722
C	Laccaria s.s. minus Laccaria ambigua	43.14	42.49	29.42-58.46	669
D		36.62	36.03	24.75-49.79	634
E		31.69	31.17	21.02-43.05	581
F	NH Laccaria	24.17	23.77	15.81-32.94	587
G	NH Laccaria w/o SE Asia	21.87	21.51	14.56-29.99	586
H		18.87	18.56	12.42-26.07	642
I		17.40	17.09	11.41-23.97	625
J	SE Asia Laccaria + China	19.94	19.60	13.14-27.51	573
K		22.39	21.97	13.79-31.31	556
L		16.27	15.68	8.38-25.09	866
M		24.49	24.05	15.41-34.25	604
N		29.16	28.63	18.80-40.63	611
O	New Zealand Clade	18.31	17.87	9.28-28.19	615
P		27.36	26.70	17.00-39.79	406

* = Node used in calibration of the dataset.
ESS = effective sample size. A value >200 is the target for effective statistical sampling.

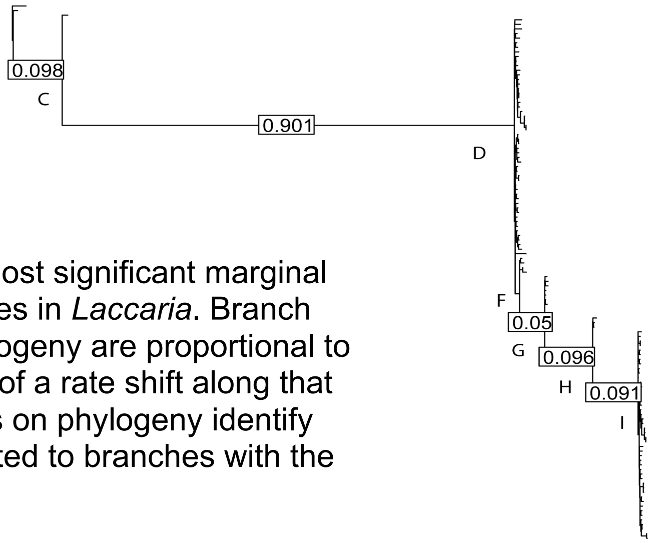

Notes S5a – Most significant marginal shift probabilities in *Laccaria*. Branch lengths in phylogeny are proportional to the probability of a rate shift along that branch. Letters on phylogeny identify nodes associated to branches with the rate shift.

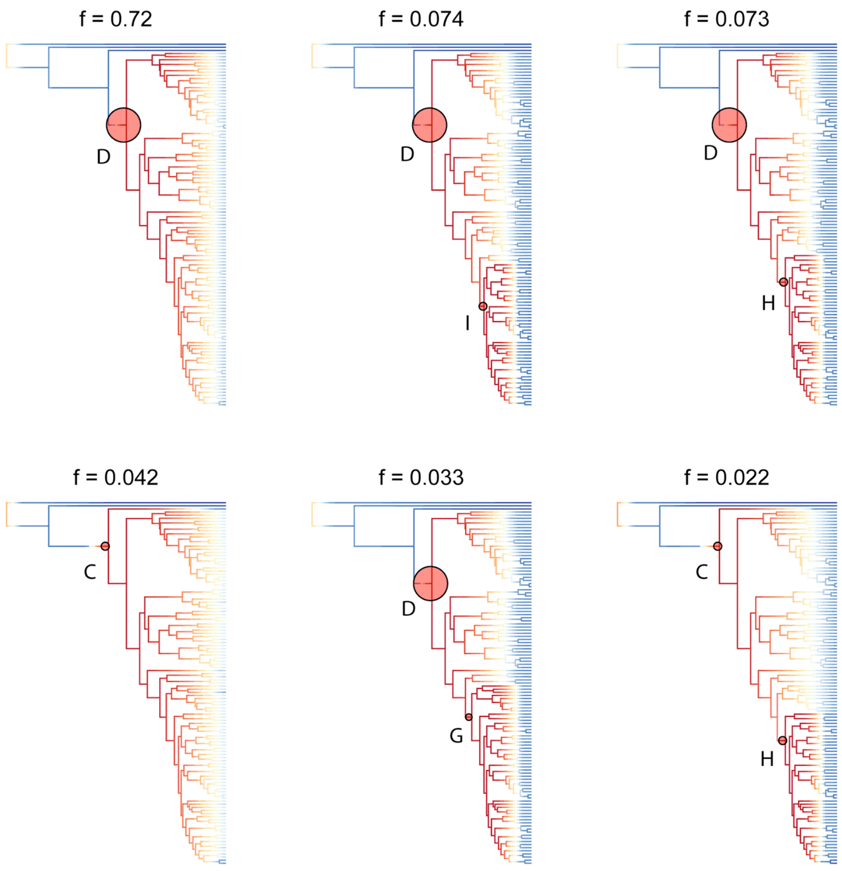

Notes S5b – 95% credible set of macroevolutionary rate configurations (CSS) in *Laccaria* based on Bayes factors (sum f > 0.95). Letters on phylogenies identify nodes associated with rate shifts.

a. BiSSE analysis: distributions of northern and southern hemisphere speciation (a1) and diversification (a2) rates.

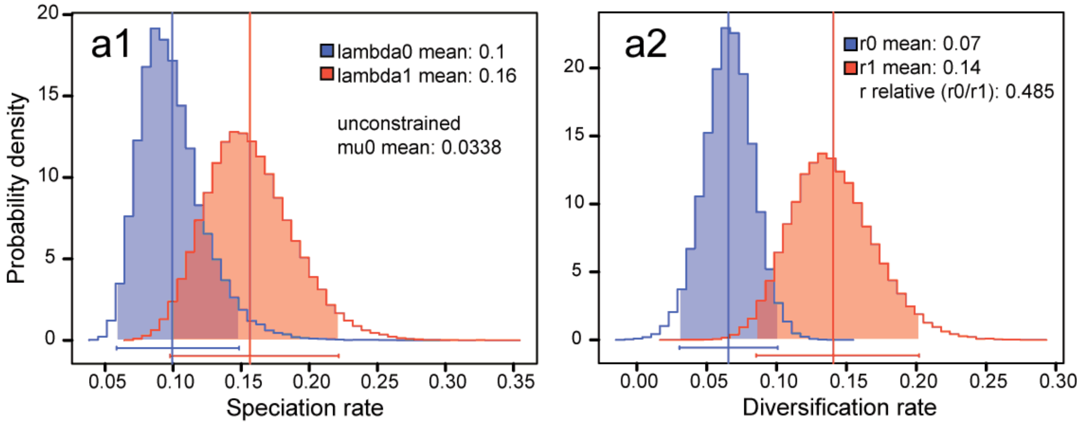

b. Likelihood ratio test between BiSSE unconstrained model and speciation (b1) or diversification (b2) null models.

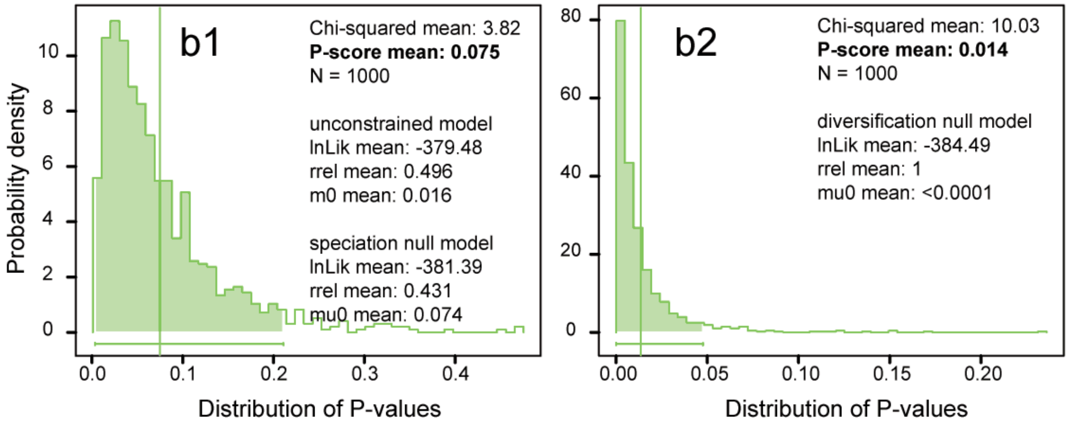

Notes S6 – a) Results of BiSSE analysis and contrasting diversification rates between northern (red) and southern (blue) hemisphere *Laccaria*. a) Northern hemisphere *Laccaria* speciation (a1) and diversification (a2) rates are elevated relative to southern hemisphere *Laccaria*. b) Likelihood ratio test using 1000 posterior sampled trees from BEAST analysis. Unconstrained speciation rates show a difference (mean P = 0.075, green vertical line) from the constrained (null) model with equal speciation rates (b1) using a P < 0.1. In contrast the model comparing diversification rates (b2) show a greater difference (mean P < 0.05) between the unconstrained versus the constrained models (mean P = 0.014).

Diversification rates (r) are measured as the difference between speciation (or lambda = λ), and extinction (or mu = μ) rates ($r = \lambda - \mu$). These rates are measured independently for two different character states representing Southern Hemisphere (state 0) and Northern Hemisphere (state 1) *Laccaria*. For example, lambda0 represents the Southern Hemisphere speciation rate, while lambda1 represent the Northern Hemisphere speciation rate. The value R_{rel} (rrel) is the relative diversification rate between the two states (R_{rel} = Southern Hemisphere diversification rate / Northern Hemisphere diversification rate = r0 / r1). An R_{rel} >1 signifies a faster Southern Hemisphere diversification rate while <1 signifies a faster Northern Hemisphere diversification rate.

Table S7 – Specimen Isotope date generated for this study

Specimen ID	Taxa	Number in figure	Site	Host	Ecology	$\delta^{13}C$	$\delta^{15}N$
PDD 89696	L. ambigua	1	Kauri Grove, Coromandel Peninsula	Leptospermum	unk	-21.96	11.56
F1080983	L. galerinoidies	2	Argentina	Nothofagus	L	-26.79	-0.51
F1081213	L. galerinoidies	3	Chile	Nothofagus	L	-26.75	4.36
PDD 89737 (KH_NZ06_119)	Laccaria NZ8	4	NZ, Mt Karioi, Waikato	Kunzea	L	-26.14	6.13
PDD 89685 (GMM7551 in tree)	Laccaria NZ4	5	NZ, Chiltern Scenic Reserve, Coromandel Peninsula	Kunzea, Leptospermum	L	-25.26	5.39
GMM7400	L Q2	6	Aus: Queensland	Allocasuarina, Eucalyptus	L	-26.43	1.11
GMM7411	L Q2	7	Aus: Queensland	Allocasuarina	L	-26.47	1.05
PDD 89677 (KH_NZ06_064)	Laccaria SpC2	8	NZ, Motutere, Coromandel Peninsula	Eucalyptus, Leptospermum	L	-25.59	5.61
GMM7316	Laccaria PNG4	9	Paupua New Guinea		L	-25.10	1.48
PDD 89812 (KH_NZ06_197)	Laccaria NZ2	10	NZ, Waitakere Regional Park, Aukland	Leptospermum, Agathis	L	-26.76	3.03
PDD 89739 (KH_NZ06_122)	Laccaria NZ3	11	NZ, Mt Karioi, Waikato	Kunzea	L	-25.30	6.01
GMM7301	L. PNG 3	12	PNG	Castanopsis, Lithocarpus	L	-26.20	3.10
GMM7302	L. PNG 3	13	PNG	Castanopsis, Lithocarpus	L	-26.10	4.24
GMM6776	Laccaria	14	China		L	-26.37	2.19
GMM7020	L. laccata s.s.	15	Russia		L	-23.33	2.16
AWW555 (SB2135 in tree)	Laccaria proxima 2	16	Magnolia Way, Nevada City, Nevada Co. CA		L	-24.11	5.43
AWW556	Laccaria	17	San Juan Ridge, Nevada City, Nevada Co. CA		L	-24.18	5.69
AWW596 (HKAS44061 in tree)	L. bicolor	18	Alaska, Highway 3, Mile 224, Carlo Creek Lodge	Picea	L	-25.96	2.02

AWW572	L. JP4	19	USA: Michigan, UP		L	-25.79	1.24
GMM7028	L. macrocystidia	20	Russia		L	-25.72	0.81
AWW628	Armillaria		13894 Magnolia Way, Nevada City, Nevada Co., CA., USA	Under Oak, Fir nearby	s	-20.85	5.96
AWW626	Boletus		13894 Magnolia Way, Nevada City, Nevada Co., CA., USA	Under Oak, Fir nearby	m	-21.51	10.11
AWW659	Boletus				m	-23.52	9.74
PDD 89655	Hydnum		Thames, Coromandel Peninsula		m	-25.90	9.47
PDD 89697	Hygrocybe		Kauri Grove, Coromandel Peninsula		unk	-25.29	6.78
PDD 89743	Hymenogaster		Mt Karioi, Waikato		m	-25.12	7.59
PDD 89672	Hyphaloma		Coromandel, Coromandel Peninsula		s	-24.69	4.28
PDD 89798	Hysterangium		Waharau Regional Park		m	-26.73	9.54
GMM6788	Leucoagaricus		China		s	-22.06	5.28
AWW647	Mycetinus		Camp Augusta, Lawn area near climbing wall, Nevada City, Nevada Co., CA	On lawn and under pine.	s	-22.86	4.89
PDD 89704	Pluteus		Kauri Grove, Coromandel Peninsula		s	-25.56	2.49
AWW656	Russula		Camp Augusta, near corrals, Nevada City, Nevada Co., CA	Under pine and cedar	m	-25.18	5.75
GMM6781	Russula		China		m	-23.63	8.51
PDD 89621	Scleroderma		Waiomu Valley, Coromandel Peninsula		m	-26.53	6.58
PDD 89703	Stephanospora		Kauri Grove, Coromandel Peninsula		unk	-22.89	9.29
PDD 89810	Tympanella		Waitakere Regional Park, Aukland		s	-22.18	1.73
PDD 89639	Weraroa		Thames, Coromandel Peninsula		s	-25.53	1.13

Abbreviations under the “Ecology” column refer to: L = *Laccaria* ECM, m = mycorrhizal, s = saprotrophic, unk = unknown.

Stable isotope abundances are reported as: $\delta^{15}N$ or $\delta^{13}C$ (‰) = $(R_{sample}/R_{standard} - 1) \times 1000$, where R is the ratio $^{13}C/^{12}C$ or $^{15}N/^{14}N$. All $\delta^{13}C$ and $\delta^{15}N$ values were normalized on VPDB ($\delta^{13}C$) and AIR ($\delta^{15}N$) reference scales with laboratory working standards of NIST 1515 (apple leaves), and tuna muscle, as well as an internal *Boletus* standard.

References

Floudas D, Binder M, Riley R, Barry K, Blanchette RA, Henrissat B, Martínez AT, Otillar R, Spatafora JW, Yadav JS, et al. 2012. The Paleozoic origin of enzymatic lignin decomposition reconstructed from 31 fungal genomes. *Science* **336**(6089): 1715-1719.

Hibbett DS, Grimaldi D, Donoghue MJ. 1997. Fossil mushrooms from Cretaceous and Miocene ambers and the evolution of homobasidiomycetes. *American Journal of Botany* **84**: 981-991.

LePage BA, Currah RS, Stockey RA, Rothwell GW. 1997. Fossil ectomycorrhizae from the Middle Eocene. *American Journal of Botany* **84**: 410-412.
